# Neuropilin-1 identifies a subset of highly activated CD8^+^ T cells during parasitic and viral infections

**DOI:** 10.1371/journal.ppat.1011837

**Published:** 2023-11-29

**Authors:** Hanna Abberger, Matthias Hose, Anne Ninnemann, Christopher Menne, Mareike Eilbrecht, Karl S. Lang, Kai Matuschewski, Robert Geffers, Josephine Herz, Jan Buer, Astrid M. Westendorf, Wiebke Hansen

**Affiliations:** 1 Institute of Medical Microbiology, University Hospital Essen, University Duisburg-Essen, Germany; 2 Division of Immunology, Walter and Eliza Hall Institute of Medical Research, Parkville, Victoria, Australia; 3 Department of Medical Biology, University of Melbourne, Parkville, Victoria, Australia; 4 Institute of Virology, University Hospital Düsseldorf, Heinrich Heine University Düsseldorf, Germany; 5 Murdoch Children’s Research Institute, Parkville, Victoria, Australia; 6 Institute of Immunology, University Hospital Essen, University Duisburg-Essen, Germany; 7 Department of Molecular Parasitology, Institute of Biology, Humboldt University Berlin, Germany; 8 Genome Analytics, Helmholtz Centre for Infection Research, Braunschweig, Germany; 9 Department of Pediatrics 1, Neonatology & Experimental perinatal Neurosciences, University Hospital Essen, University Duisburg-Essen, Germany; 10 Centre for Translational Neuro- and Behavioral Sciences, C-TNBS, Faculty of Medicine, University Duisburg-Essen, Germany; Indiana University School of Medicine, UNITED STATES

## Abstract

Neuropilin-1 (Nrp-1) expression on CD8^+^ T cells has been identified in tumor-infiltrating lymphocytes and in persistent murine gamma-herpes virus infections, where it interferes with the development of long-lived memory T cell responses. In parasitic and acute viral infections, the role of Nrp-1 expression on CD8^+^ T cells remains unclear. Here, we demonstrate a strong induction of Nrp-1 expression on CD8^+^ T cells in *Plasmodium berghei* ANKA (PbA)-infected mice that correlated with neurological deficits of experimental cerebral malaria (ECM). Likewise, the frequency of Nrp-1^+^CD8^+^ T cells was significantly elevated and correlated with liver damage in the acute phase of lymphocytic choriomeningitis virus (LCMV) infection. Transcriptomic and flow cytometric analyses revealed a highly activated phenotype of Nrp-1^+^CD8^+^ T cells from infected mice. Correspondingly, *in vitro* experiments showed rapid induction of Nrp-1 expression on CD8^+^ T cells after stimulation in conjunction with increased expression of activation-associated molecules. Strikingly, T cell-specific Nrp-1 ablation resulted in reduced numbers of activated T cells in the brain of PbA-infected mice as well as in spleen and liver of LCMV-infected mice and alleviated the severity of ECM and LCMV-induced liver pathology. Mechanistically, we identified reduced blood-brain barrier leakage associated with reduced parasite sequestration in the brain of PbA-infected mice with T cell-specific Nrp-1 deficiency. In conclusion, Nrp-1 expression on CD8^+^ T cells represents a very early activation marker that exacerbates deleterious CD8^+^ T cell responses during both, parasitic PbA and acute LCMV infections.

## Author summary

Malaria is caused by the parasite *Plasmodium* and remains one of the most life threatening infectious diseases worldwide. In 2021, WHO reported 247 million malaria cases and more than 600,000 deaths. In this study, we infect mice with *Plasmodium berghei* ANKA (PbA), a scientific model of experimental cerebral malaria (ECM), which affects the brain and is often fatal. During infection, CD8^+^ T cells are known to invade the brain and contribute to brain damage and inflammation through their activated and cytotoxic potential. We investigate whether T cell-expressed Neuropilin-1 (Nrp-1), a co-receptor for semaphorin and vascular endothelial growth factor (VEGF) known to play a role in immune cell activation and migration, has an impact on ECM manifestation. The results of our study demonstrate that Nrp-1 is associated with increased T cell activation and significantly aggravates the severity of ECM. Importantly, our results are also applicable to other CD8^+^ T cell-induced immunopathologies as we show similar effects during lymphocytic choriomeningitis virus (LCMV) infection. Together, this implies that Nrp-1 may be an early prognostic marker and blockade of Nrp-1 could be a promising target to control exacerbated T cell responses in different diseases.

## Introduction

The glycoprotein Neuropilin-1 (Nrp-1) was originally discovered during neuronal development and angiogenesis [1, 2], but is also involved in immune cell activation and migration [3–5]. Nrp-1 is predominantly expressed by endothelial, tumor and immune cells. Among the latter, Nrp-1 is expressed by macrophages, dendritic cells, mast cells and CD4^+^ T cells, in particular, by CD4^+^Foxp3^+^ regulatory T cells (Tregs) [6–8]. It serves as a co-receptor for several ligands, including vascular endothelial growth factor (VEGF), semaphorins and TGF-β [8]. The recent identification of Nrp-1 as co-receptor for SARS-CoV-2 entry reaffirms its central role in numerous important cellular processes [9, 10]. Moreover, homophilic interaction of Nrp-1 molecules is involved in T cell activation as it increases the duration of immune cell contact and stabilizes the immunological synapse between T cells and DCs [3, 4].

With regard to T cells, the role of Nrp-1 is most comprehensively defined in the Treg population. Within this group, Nrp-1 expression is known to correlate with the expression of the transcription factor Foxp3, which is important for the stability of these cells [7, 11]. In experimental autoimmune encephalomyelitis (EAE), a model of the chronic neuroinflammatory disorder multiple sclerosis, the suppressive function of Tregs was decreased in mice lacking Nrp-1 expression on T cells, resulting in exacerbation of EAE [12]. Previously, we showed that the Nrp-1 co-receptor on Tregs plays an essential role in Treg migration [5]. In melanoma, Nrp-1^+^ Tregs migrate along a VEGF gradient into the tumor tissue and suppress anti-tumoral immune responses [5]. Treg-specific Nrp-1 depletion resulted in less stability and function of intratumoral Tregs and a decrease in tumor volume [11]. Alongside Tregs, of which the majority express Nrp-1, we recently demonstrated that CD4^+^Foxp3^-^CD25^-^ conventional T cells also strongly induce Nrp-1 expression upon *in vitro* and *in vivo* stimulation [13]. These Nrp-1^+^CD4^+^ effector T cells are highly activated compared to their Nrp-1^-^ counterparts. In accordance, Gaddis and colleagues observed an increased frequency of Nrp-1^+^CD4^+^ effector T cells in a model of atherosclerosis that was also associated with an activated phenotype and ablation of Nrp-1 on T cells led to a better disease outcome [14].

In their naïve state, CD8^+^ T cells display low levels of Nrp-1 expression, which are significantly elevated during persistent gamma-herpes virus infection, autoimmune diseases and cancer [15–18]. Intra-tumoral Nrp-1^+^CD8^+^ T cells are reported to co-express molecules such as PD-1, Tim-3 and CTLA-4 and appear to be impaired in their migratory and effector functions [15, 18]. However, ablation of Nrp-1 expression on CD8^+^ T cells did not affect acute tumor growth in mice. Nevertheless, it is debated whether this would impair the development of T cell memory [18]. CD8^+^ T cell-specific Nrp-1 knockout mice with a resection of the primary tumor were protected from secondary tumor challenge. Consequently, Nrp-1 has been proposed as an immune checkpoint molecule on CD8^+^ T cells in cancer [15]. The expression and role of Nrp-1 on CD8^+^ T cells during parasitic and acute viral infections remain elusive.

Malaria, caused by the parasite *Plasmodium* (*P*.), is still one of the most widespread and deadly human infectious diseases worldwide [19]. Infection with *P*. *falciparum* may result in the development of cerebral malaria, the most severe neurological complication of *Plasmodium* infection with a high mortality rate [20]. To study the molecular and immunological processes during cerebral malaria, the use of PbA-infected C57BL/6 mice as a rodent model is well established [21]. CD8^+^ T cells play the major role in the manifestation of experimental cerebral malaria (ECM) [22, 23]. Upon parasite accumulation in the blood, the innate immune response is triggered and systemic IFN-γ and TNF-α levels are elevated. In the brain, infected erythrocytes attach to the endothelium, leading to a further increase in chemokine and cytokine levels, which activate endothelial cells that uniquely perform cross-presentation of parasite antigens [23]. Activated CD8^+^ T cells from the periphery migrate to the brain, recognize *Plasmodium* antigens and kill the antigen-presenting endothelial cells by releasing perforin and granzyme B (GzmB) [24, 25]. Destabilization of the blood-brain barrier facilitates an influx of peripheral immune cells into the brain [23]. Subsequent neuroinflammation may develop into cerebral malaria associated with severe neurological deficits. Thus, a well-balanced immune response is essential to eliminate the pathogen without inducing a severe immunopathology. This balance is not only relevant in parasitic infections, but also in viral infections. A well-established model system for viral infection in mice is the lymphocytic choriomeningitis virus (LCMV). LCMV is a single-stranded RNA virus that belongs to the Arenaviridae family. The virus itself has no cytopathic effects, however the activation and cytotoxic function of CD8^+^ T cells required to clear LCMV can be associated with pathological damage to the host [26–28].

In the present study, we investigated the role of Nrp-1 expression on CD8^+^ T cells during parasitic and acute viral infections. We infected C57BL/6 mice with PbA or LCMV strain WE (LCMV-WE) and analyzed the T cell phenotype and the influence of Nrp-1 expression on manifestation of ECM and liver damage during acute viral infection. Moreover, we characterized Nrp-1^+^ and Nrp-1^-^CD8^+^ T cells by transcriptome analysis and flow cytometry and analyzed the function of Nrp-1 expression *in vitro*.

## Results

### *Plasmodium berghei* ANKA infection induces Nrp-1 expression on CD8^+^ T cells that correlates with disease severity

**Fig 1:**
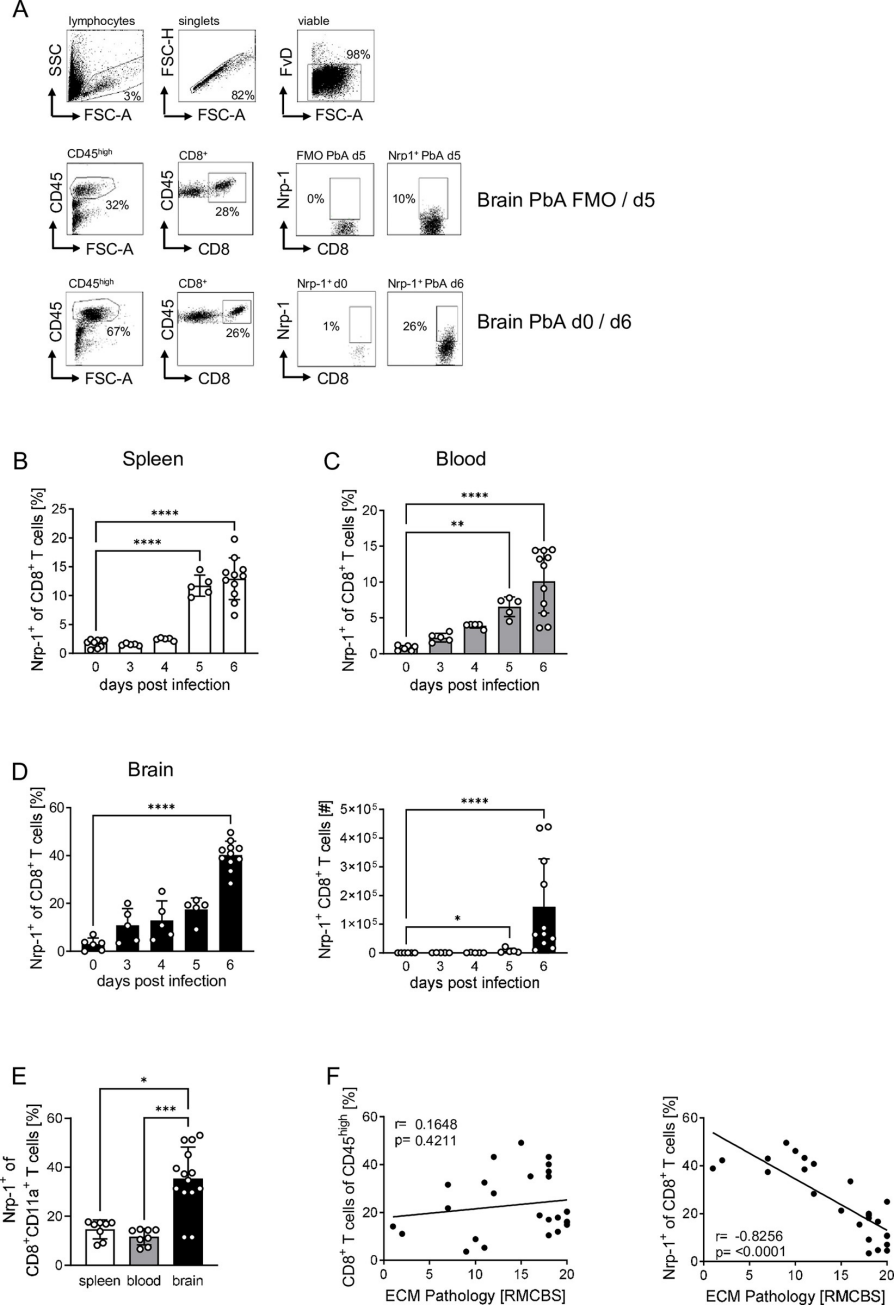
*Plasmodium berghei ANKA* infection induces Nrp-1 expression on CD8^+^ T cells that correlates with disease severity. Nrp-1 expression on CD8^+^ T cells was analyzed by flow cytometry in naïve (0 days post infection) and PbA-infected C57BL/6 mice at day 3, 4, 5 and 6 post infection. (A) A representative gating strategy, including fluorescence minus one (FMO) control, is shown for immune cells in the brain 5 days after PbA infection or from non-infected (d0) and PbA-infected mice (d6). The frequency of Nrp-1-expressing CD8^+^ T cells was analyzed in (B) spleen, (C) blood and (D, left) brain. (D, right) Absolute numbers of Nrp-1^+^CD8^+^ T cells are shown for the brain. (E) The percentages of Nrp-1 expressing cells among gated CD8^+^CD11^+^ antigen-experienced T cells were analyzed in spleen, blood and brain 6 days post PbA infection by flow cytometry. (F) Correlation was calculated between the frequency of cerebral CD8^+^ T cells (left) or Nrp-1^+^CD8^+^ T cells (right) during the course of PbA infection and the severity of experimental cerebral malaria (ECM) pathology assessed by the Rapid-Murine-Coma-and-Behavior-Scale (RMCBS) score. ECM pathology characterized by neurologic deficits results in a decline in score with increasing severity. (B-D) Results from one (day 3, 4, 5) or two (day 0 and 6) experiments with n = 5–11 mice per time point are summarized as mean (± SD). (E) Results from two experiments with n = 8–14 mice in total are shown as mean. Each dot represents one animal. Statistical analysis was performed with (C, D, E) nonparametric Kruskal-Wallis test and Dunn’s multiple comparisons test and with (B) ordinary one-way ANOVA and Holm-Sidak’s multiple comparisons test. *, p<0.05; **, p<0.01; ***, p<0.001, ****, p<0.0001. (F) Correlation is shown based on n = 26 mice and statistical significance was calculated using nonparametric Spearman correlation test. P and r values are displayed in the graphs.

To investigate the role of Nrp-1 in CD8^+^ effector T cells during malaria, we first analyzed Nrp-1 expression on CD8^+^ T cells during PbA infection in C57BL/6 mice via flow cytometry (Fig 1A). In spleen, blood and brain of naïve mice, Nrp-1 expression on the surface of CD8^+^ T cells was almost absent (< 2% 0 days post infection, Fig 1B–1D). Following PbA infection, the proportion of Nrp-1-expressing CD8^+^ T cells in the spleen and blood significantly increased from day 5 onwards (Fig 1B and 1C). On day 5, we first observed neurological deficits and the strong activation of CD8^+^ T cells (S1A and S1C Fig). ECM manifestation was observed on day 6 (S1A and S1B Fig) and 13% of CD8^+^ T cells in the spleen and 10% of circulating CD8^+^ T cells in the peripheral blood expressed Nrp-1 on the surface (Fig 1B and 1C). In the brain of PbA-infected C57BL/6 mice, progression of cerebral malaria led to a striking increase in Nrp-1 expression on CD8^+^ T cells between day 5 and 6: Approximately 40% of CD8^+^ T cells expressed Nrp-1 (Fig 1D, left). In absolute numbers, Nrp-1^+^CD8^+^ T cells increased by a thousand-fold from 110 in the brains of naïve mice to approximately 160,000 Nrp-1^+^CD8^+^ T cells in the damaged brain of PbA-infected mice (Fig 1D, right). In addition, we detected elevated frequencies of Nrp-1-expressing CD8^+^CD11a^+^ antigen-experienced T cells in the brain compared to CD8^+^CD11a^+^ T cells in the spleen and blood of PbA-infected mice at 6 days post infection (Fig 1E).

Given the timing of the accumulation of Nrp-1^+^CD8^+^ T cells in the brain, we investigated whether the proportion of Nrp-1^+^CD8^+^ T cells in the brain correlates with the severity of cerebral malaria. ECM symptoms, including a range of neurological and behavioral deficits, were quantified using the well-established rapid murine coma and behavior scale (RMCBS) score [29]. Healthy animals typically have a score of 18–20. As disease progresses, the RMCBS score decreases and animals with a score below 12 are considered to have ECM (S1A Fig). As shown in Fig 1F, the frequency of total CD8^+^ T cells in the brain was independent of the RMCBS score (left), but the frequency of Nrp-1^+^CD8^+^ T cells significantly correlated with the severity of ECM (right). The more advanced the ECM manifestation, the higher the percentage of Nrp-1-expressing CD8^+^ T cells in the brain of PbA-infected C57BL/6 mice (Fig 1F). For comparison, we also examined Nrp-1 expression on Foxp3^-^ and Foxp3^+^CD4^+^ T cells (S2 Fig). As reported, the majority of Foxp3^+^ Tregs in the spleen [7] and blood expressed Nrp-1. Upon PbA infection, we detected a slight decrease of the proportion of Nrp-1-expressing Tregs in the spleen and peripheral blood. The percentage of Nrp-1-expressing conventional CD4^+^ T cells increased considerably in all organs tested, although to a much lesser extent than that of Nrp-1^+^CD8^+^ T cells (S2 Fig).

Together, we demonstrated a strong induction of Nrp-1 expression on brain-infiltrating CD8^+^ T cells and to a lesser extent on conventional CD4^+^ T cells during the progression of PbA infection and with the onset of neurological deficits associated with ECM.

### Nrp-1^+^CD8^+^ T cells display a highly activated phenotype during ECM

**Fig 2:**
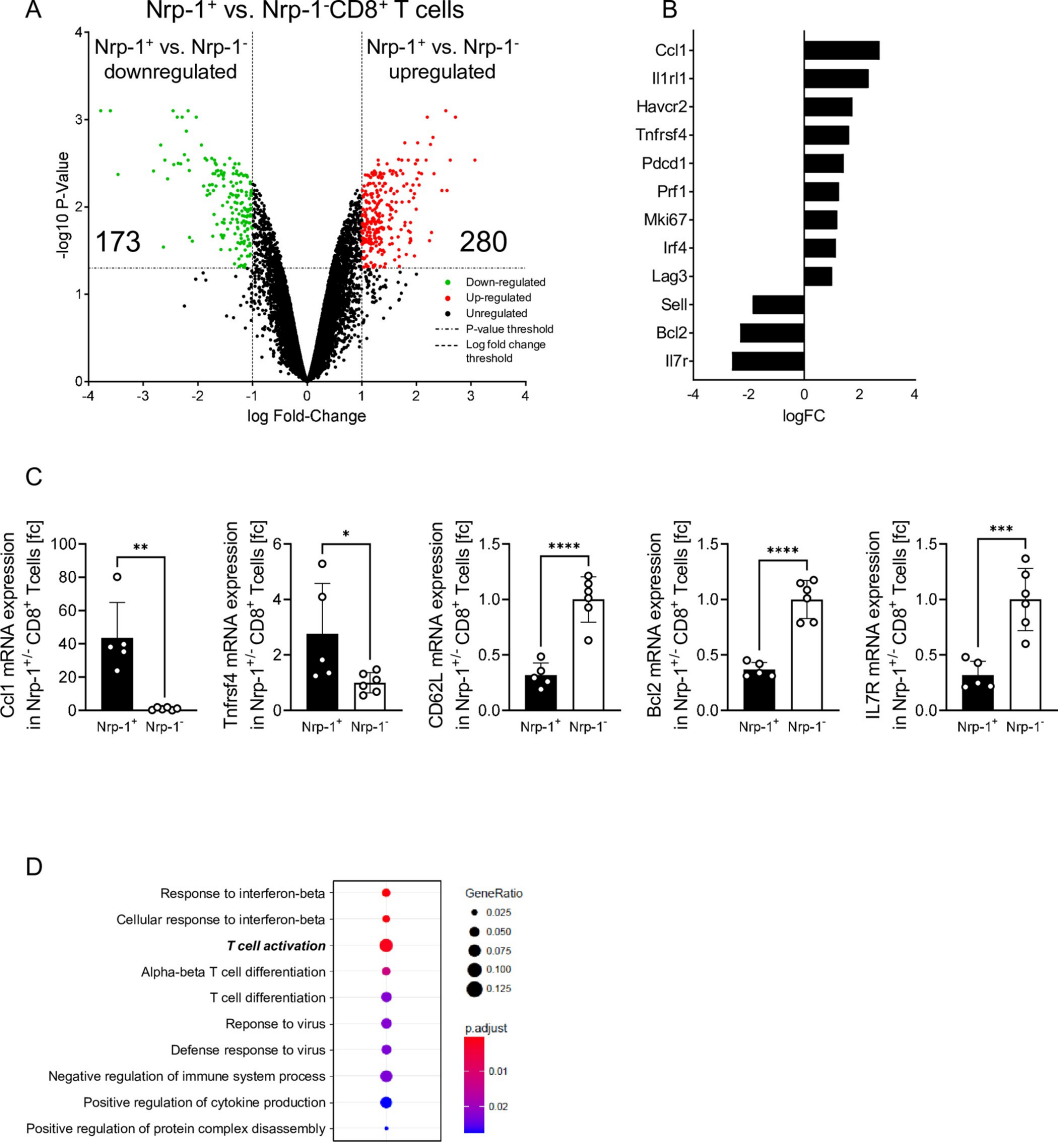
Differential gene expression analysis reveals differences in T cell activation. For analysis of differentially expressed genes, Nrp-1^+^ and Nrp-1^-^CD8^+^ T cells were sorted from spleens of PbA-infected C57BL/6 mice at day 6 post infection (n = 2–3), processed and analyzed by Clariom S microarray analysis. (A) The volcano plot summarizes 173 genes significantly downregulated (colored green) and 280 genes significantly upregulated (colored red) in Nrp-1^+^ compared to Nrp-1^-^ CD8^+^ T cells. (B) Differential gene expression of selected genes of Nrp-1^+^CD8^+^ vs. Nrp-1^-^CD8^+^ T cells. (C) mRNA expression of selected genes was analyzed in sorted Nrp-1^+^CD8^+^ vs. Nrp-1^-^CD8^+^ T cells from spleens of C57BL/6 mice 6 days post PbA infection by qRT-PCR (n = 5 mice, each dot represents the mean of technical duplicates from one mouse). Data are summarized as mean ± SD and Student`s t-test was used for statistical analysis. *, p<0.05; **, p<0.01; ***, p<0.001; ****, p<0.0001. (D) Enriched Gene Ontology Analysis was performed with GO-Term “Biological Process”. The gene ratio increased with perimeter and the adjusted p-value is shown as high in blue and low in red color of the dots.

As CD8^+^ T cells play a crucial role during ECM, we characterized the phenotypes of Nrp-1^+^ and Nrp-1^-^ CD8^+^ T cells during ECM in more detail. Therefore, we sorted Nrp-1^+^ and Nrp-1^-^ CD8^+^ T cells from spleens of PbA-infected C57BL/6 mice 6 days after infection and analyzed the gene expression profile by Clariom S microarray analysis. The volcano plot in Fig 2A summarizes 173 genes that were significantly downregulated in Nrp-1^+^ compared to Nrp-1^-^CD8^+^ T cells and 280 genes that were upregulated. A selection of regulated genes associated with immune cell function is depicted in Fig 2B. To confirm results from our microarray analysis, we determined the mRNA expression levels of Ccl1, Tnfrsf4, CD62L (*Sell*), Bcl2 and IL7R in sorted Nrp-1^+^ and Nrp-1^-^ CD8^+^ T cells from the spleen of PbA-infected C57BL/6 mice by qPCR (Fig 2C). Interestingly, genes associated with immune checkpoints, such as *Havcr2* (Tim-3), *Pdcd1* and *Lag3* [30, 31], were more strongly expressed by Nrp-1^+^CD8^+^ compared to Nrp-1^-^CD8^+^ T cells (Fig 2B). Expression of the genes *Prf1*, which is related to cytotoxic CD8^+^ T cell function, *Mki67*, which is involved in proliferation, and *Ccl1*, which is associated with cell migration, were increased in Nrp-1^+^ CD8^+^ T cells compared to Nrp-1^-^ CD8^+^ T cells. *Il1rl1*, *Irf4* and *Tnfrsf4* (OX40), which are known to play a role in effector T cell activation, differentiation and function [32–34] were enhanced in Nrp-1^+^ cells. Accordingly, *Sell* (L-selectin or CD62L) was reduced in Nrp-1^+^CD8^+^ T cells, which is also associated with increased T cell activation [35]. *Bcl2* and *Il7r* (CD127), both genes that are known to be important as anti-apoptotic factors, [36] were downregulated in Nrp-1^+^CD8^+^ T cells as opposed to Nrp-1^-^ counterparts. Hence, Nrp-1^+^CD8^+^ T cells seem to be strongly activated cells, as genes associated with effector functions were upregulated in contrast to Nrp-1^-^CD8^+^ T cells. Indeed, enriched gene ontology analysis revealed that genes involved in the cellular response to cytokines, viral infection or T cell differentiation were differentially expressed by Nrp-1^+^ and Nrp-1^-^ CD8^+^ T cells from PbA-infected mice. In particular, the expression of several genes important for T cell activation was significantly different between the groups associated with a relatively high geneRatio and a low adjusted p value shown in red (Fig 2D).

**Fig 3:**
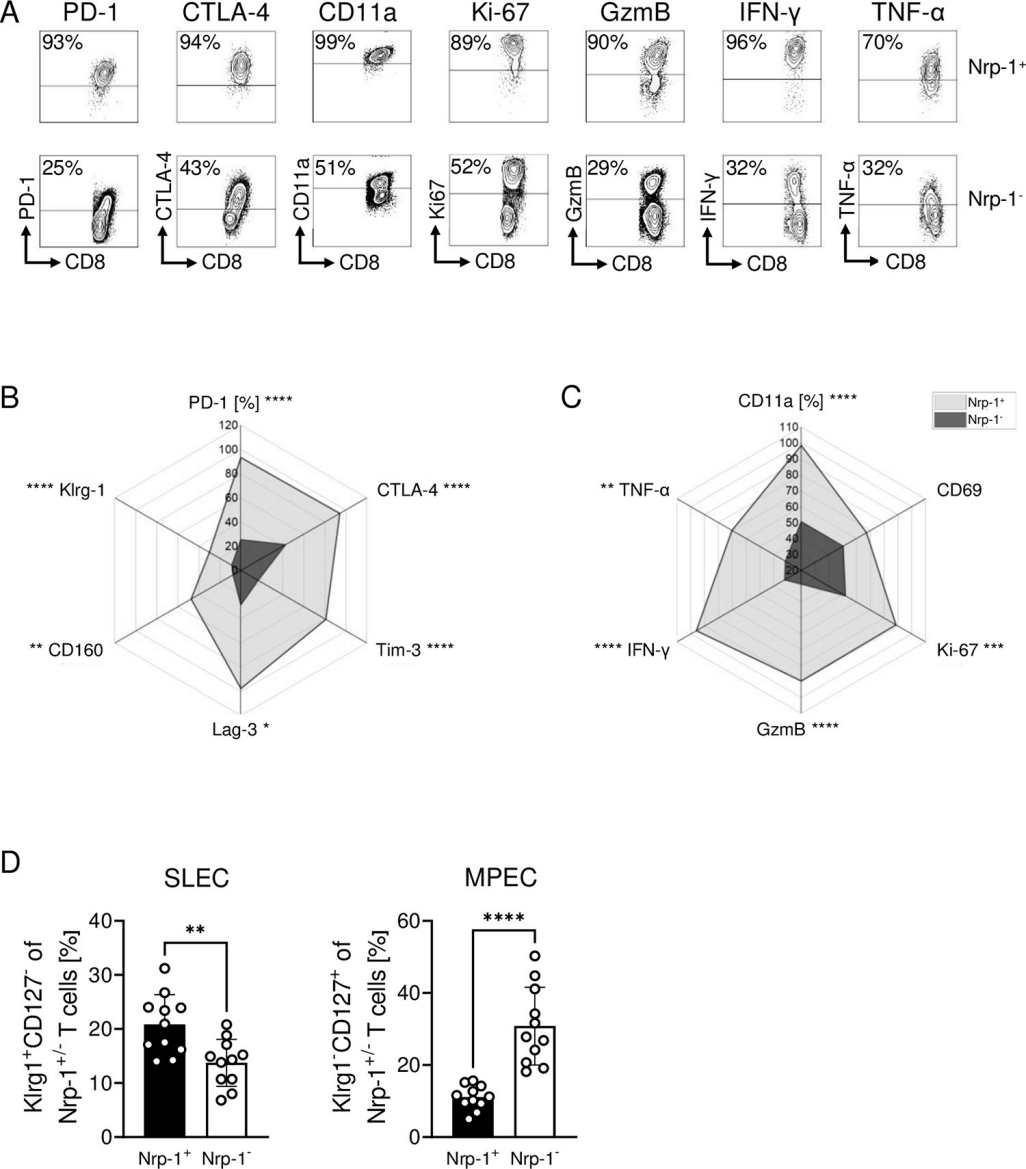
Nrp-1^+^CD8^+^ T cells display a highly activated phenotype during ECM. (A) Representative contour plots show the expression of activation-associated molecules of Nrp-1^+^ (top row) and Nrp-1^-^ CD8^+^ T cells (bottom row) analyzed by flow cytometry in the spleen of PbA-infected C57BL/6 mice 6 or 7 days after infection. (B and C) Radiation plots summarize the mean expression of malaria-associated molecules on Nrp-1^+^ (grey area) and Nrp-1^-^ CD8^+^ T cells (black area) measured by flow cytometry. Data from one experiment with n = 3–5 mice are shown. Mann-Whitney test was used to calculate statistics. (D) The percentages of Klrg-1^+^CD127^-^ short-lived effector cells (SLEC) and Klrg-1^-^CD127^+^ memory-precursor effector cells (MPEC) among Nrp-1^+^CD8^+^ and Nrp-1^-^CD8^+^ T cells from spleen of PbA-infected C57BL/6 mice were analyzed by flow cytometry. Data from two independent experiments with n = 10–11 mice in total are summarized as mean ± SD. Student´s t-test was used for statistical analysis *, p<0.05; **, p<0.01; ***, p<0.001; ****, p<0.0001.

We further confirmed a high activation state of Nrp-1^+^CD8^+^ T cells in comparison to Nrp-1^-^CD8^+^ T cells from the spleen of PbA-infected mice by flow cytometry (Fig 3A). Nrp-1-expressing CD8^+^ T cells were associated with a strong protein expression of PD-1, CTLA-4, Tim-3, Lag-3, CD160 and Klrg-1 (Fig 3B). Moreover, in contrast to Nrp-1^-^CD8^+^ T cells, Nrp-1^+^CD8^+^ T cells showed a significantly increased protein expression of molecules associated with antigen-experience (CD11a), early T cell activation (CD69), proliferation (Ki-67), cytotoxicity (GzmB) and cytokines related to ECM pathology (IFN-γ and TNF-α) (Fig 3C). In addition, we detected higher percentages of short-lived effector cells (SLEC) and lower frequencies of memory precursor effector cells (MPEC) among Nrp-1^+^CD8^+^ T cells compared to Nrp-1^-^CD8^+^ T cells, as determined by the expression of Klrg-1 and CD127 (Fig 3D). In summary, PbA infection induced a Nrp-1^+^CD8^+^ T cell subset associated with high expression of effector molecules characteristic for *Plasmodium* infection and associated disease [23].

### Nrp-1 expression defines a highly activated CD8^+^ T cell phenotype *in vitro*

**Fig 4:**
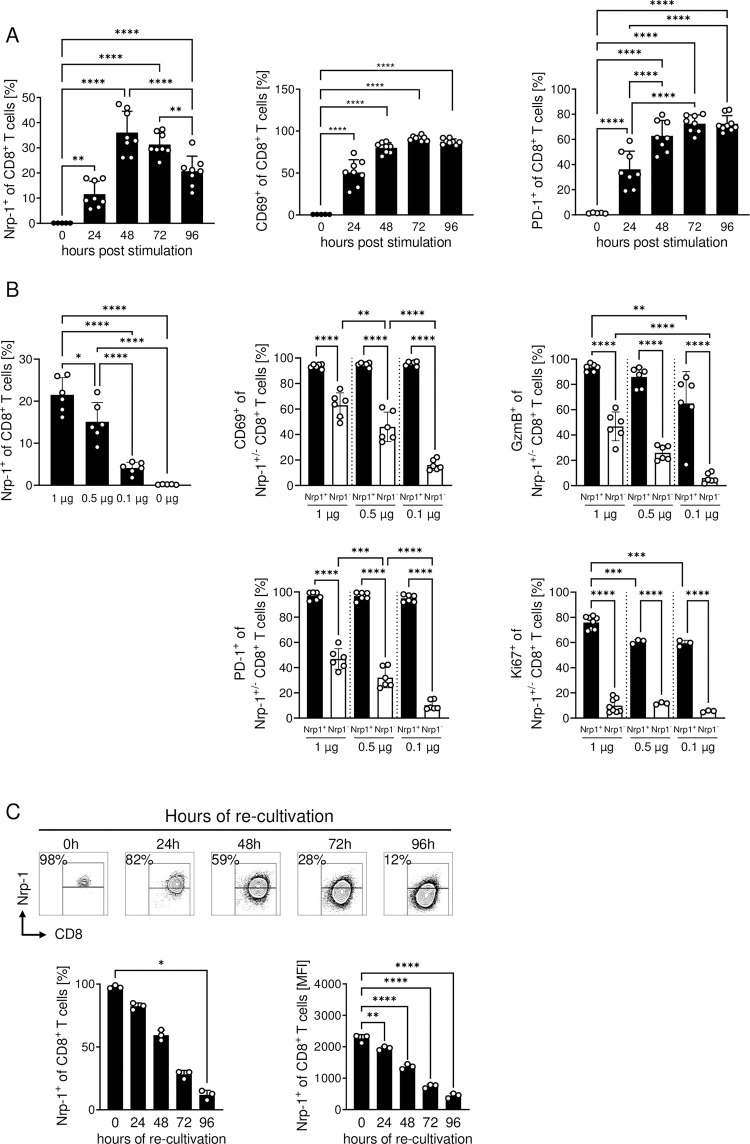
Nrp-1 expression defines a highly activated CD8^+^ T cell phenotype. Splenic CD8^+^ T cells from naïve C57BL/6 mice were stimulated with αCD3 and αCD28 *in vitro*. (A) Nrp-1, CD69 and PD-1 expression of CD8^+^ T cells was analyzed by flow cytometry after 0, 24, 48, 72 or 96 hours of *in vitro* stimulation with 1 μg/ml αCD3/αCD28. (B) Nrp-1 expression on gated CD8^+^ T cells (left) and the expression of activation-associated molecules was analyzed by flow cytometry on Nrp-1^+^ (black bars) and Nrp-1^-^ CD8^+^ T cells (white bars) 48 hours after *in vitro* stimulation with 1 μg/ml, 0.5 μg/ml or 0.1 μg/ml αCD3/αCD28 and is summarized as mean ± SD. Experiments have been performed as technical duplicates of stimulated T cells from one mouse. Mean values of duplicates have been included as one data point that represents one biological replicate (cells from one mouse). (C) The stability of Nrp-1 expression after *in vitro* induction was analyzed by isolating Nrp-1^+^CD8^+^ T cells using FACS after 48 hours of stimulation. The cells were re-cultured with IL-2 and Nrp-1 expression was analyzed at 0, 24, 48, 72 and 96 hours after re-cultivation by flow cytometry as shown by exemplary contour plots. Data from one to two independent experiments with (A) n = 8 mice in total and (B) n = 3–8 mice in total and (C) one experiment with n = 3 mice in total are presented as mean values with SD. (A, B) Ordinary one-way ANOVA with Holm-Sidak’s or Tukey’s multiple comparisons test or (C) Kruskal-Wallis test with Dunn’s multiple comparisons test were used to test significance. *, p<0.05; **, p<0.01; ***, p<0.001; ****, p<0.0001.

To gain further insights into the function of Nrp-1 expression on CD8^+^ T cells in general, we analyzed these cells in more detail *in vitro* (Fig 4). While unstimulated splenic CD8^+^ T cells from naïve C57BL/6 mice generally lacked Nrp-1 expression (mean 0.1%), *in vitro* stimulation with 1 μg αCD3/αCD28 resulted in a highly significant increase in Nrp-1-expressing CD8^+^ T cells with a maximum of 36% Nrp-1^+^CD8^+^ T cells after 48 hours of stimulation (Fig 4A). After this time point, the frequency of Nrp-1^+^CD8^+^ T cells decreased, which was clearly different from classical activation markers such as CD69 and PD-1. The expression of CD69 and PD-1 was rapidly induced during *in vitro* stimulation and was stable until at least 96 hours of cultivation (Fig 4A). Next, we stimulated MACS-sorted CD8^+^ T cells from spleen of C57BL/6 mice with 1 μg/ml αCD3/αCD28, 0.5 μg/ml αCD3/αCD28 or 0.1 μg/ml αCD3/αCD28 *in vitro*. After two days we analyzed the expression of Nrp-1 on gated CD8^+^ T cells (Fig 4B, left) and the expression of CD69, PD-1, GzmB and Ki-67 on gated Nrp-1^+^CD8^+^ T cells and Nrp-1^-^CD8^+^ T cells by flow cytometry (Fig 4B). Consistent with our *in vivo* results from PbA-infected mice (Figs 2 and 3), flow cytometric analyses revealed a highly activated phenotype for *in vitro*-generated Nrp-1^+^CD8^+^ T cells, even when stimulated with low concentrations of αCD3/αCD28 (0.1 μg/ml). Nrp-1^+^CD8^+^ T cells strongly expressed CD69 and PD-1, showed increased proliferation (Ki-67) and enhanced cytotoxic function (GzmB) compared to CD8^+^ T cells lacking Nrp-1 expression (Fig 4B). We obtained similar results also for antigen-specific CD8^+^ T cells stimulated with the cognate antigen in the presence of irradiated splenocytes as antigen presenting cells. Stimulation of OVA (Ovalbumin)-specific T cells with different concentrations of OVA for 48 hours resulted in enhanced Nrp-1 expression (S3A Fig). CD69- and PD-1-expressing cells were again elevated among Nrp-1^+^CD8^+^ OVA-specific T cells compared to Nrp-1^-^CD8^+^ OVA-specific T cells (S3B Fig).

**Fig 5:**
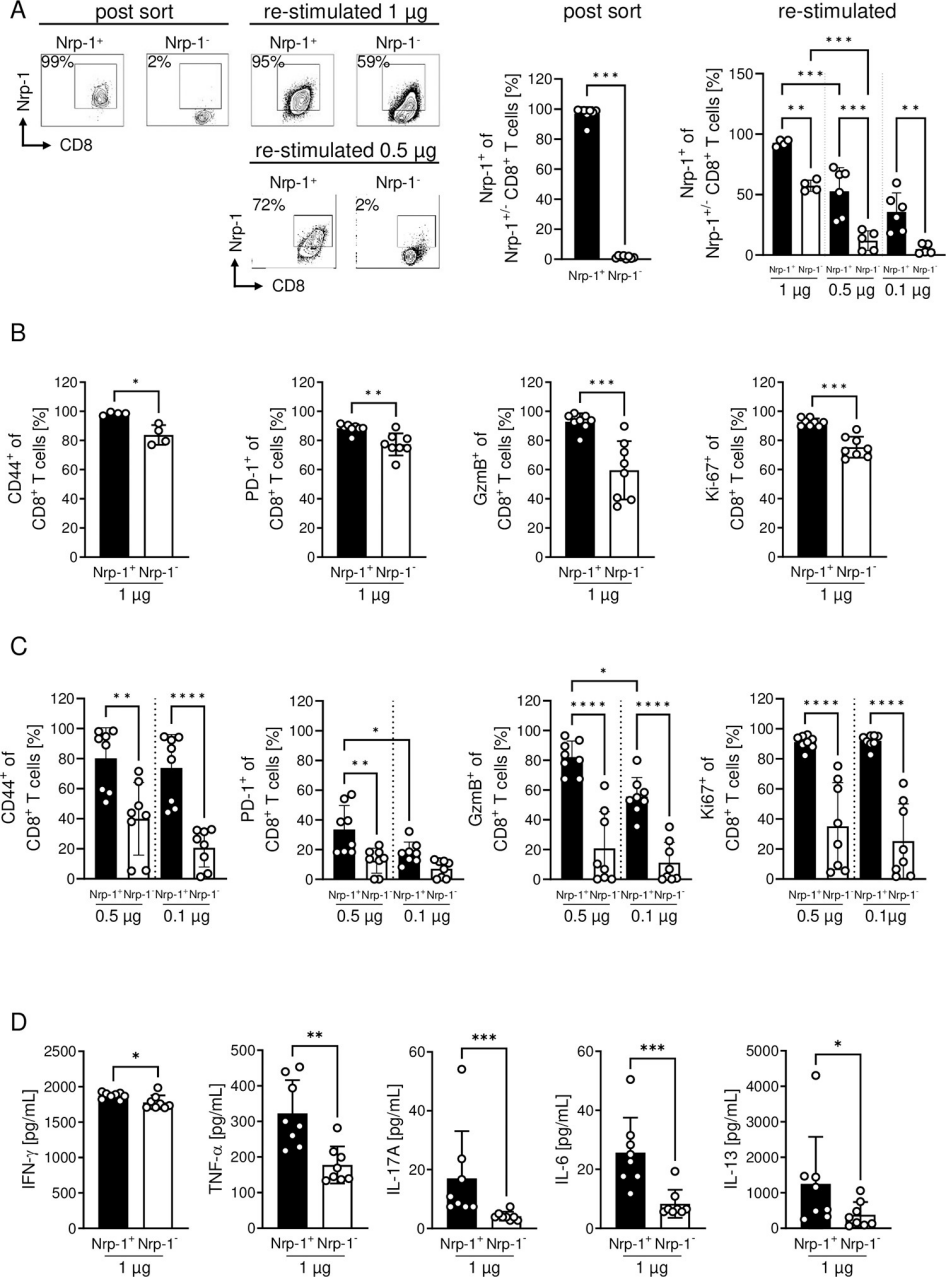
Re-stimulated Nrp-1^+^CD8^+^ T cells maintained their high level of T cell activation. CD8^+^ T cells from Nrp-1tdTomato reporter mice or from C57BL/6 mice were separated into Nrp-1tdtomato^+^ or Nrp-1^+^ fluorochrome-labeled antibody-stained (represented as black bars) and Nrp-1^-^ not endogenously expressing tdTomato or negative for Nrp-1 fluorochrome-labeled antibody staining (white bars) by FACS sorting 48 hours after *in vitro* stimulation. Cells were then stimulated again with αCD3 and αCD28 for 48 hours and analyzed by flow cytometry. (A) Nrp-1 expression of Nrp-1^+^ or Nrp-1^-^ CD8^+^ T cells after sorting (post-sort) and re-stimulation with indicated concentrations of αCD3 and αCD28 was measured by flow cytometry. (B and C) The activation state (CD44, PD-1), effector function (GzmB) and proliferation (Ki-67) of CD8^+^ T cells that were either Nrp-1^+^ or Nrp-1^-^ prior to re-stimulation was analyzed 48 hours after re-stimulation with (B) 1 μg/ml, (C) 0.5 μg/ml or 0.1 μg/ml αCD3/αCD28 on gated CD8^+^ T cells by flow cytometry. (D) The supernatants of this re-cultivation (with 1 ug/ml αCD3/αCD28) were collected and the concentrations of cytokines were determined using Luminex technology. Data from two to three independent experiments with n = 5–8 mice in total are shown as mean ± SD. Depending on the sorted cell number of Nrp-1^+^ or Nrp-1^-^ CD8^+^ T cells, some of the experiments have been performed as technical duplicates. In this case, the mean was calculated and plotted as one dot. (A, C) ordinary one-way ANOVA with Holm-Sidak’s multiple comparisons test, (B) Student’s t-test or (D) Mann-Whitney test were used to test significance. *, p<0.05; **, p<0.01; ***, p<0.001; ****, p<0.0001.

In further experiments, we examined the stability of Nrp-1 expression on FACS cell-sorted Nrp-1^+^CD8^+^ T cells from WT mice generated by 48 hour *in vitro* stimulation by re-culturing them in the presence of IL-2. Within 96 hours after re-culturing without T cell receptor (TCR) stimulus, Nrp-1^+^CD8^+^ T cells continually lost Nrp-1 expression (Fig 4C). However, when *in vitro* generated (48 hours after stimulation) and sorted Nrp-1^+^ and Nrp-1^-^ CD8^+^ T cells (Fig 5A) were re-stimulated with 1 μg/ml αCD3 and αCD28, Nrp-1 expression was stable for at least another 48 hours, but declined after stimulation with 0.5 μg/ml or 0.1 μg/ml αCD3 and αCD28 (Fig 5A). Thus, it appears that upregulation of Nrp-1 expression on CD8^+^ T cells is a rapid mechanism that requires a constant and strong activation stimulus.

Strikingly, the activation state (analyzed by CD44 and PD-1 expression), proliferation (determined by Ki-67 expression) and expression of the effector molecule GzmB remained higher in re-stimulated Nrp-1^+^CD8^+^ T cells compared to re-stimulated Nrp-1^-^CD8^+^ T cells (Fig 5B). These differences were even more pronounced after stimulation with lower concentrations of αCD3 and αCD28 (0.5 μg/ml and 0.1/ml μg/ml) (Fig 5C). Quantification of cytokines from the supernatants of Nrp-1^+^CD8^+^ T cells re-stimulated with 1 μg/ml αCD3 and αCD28 for 48h showed increased concentrations of IFN-γ, TNF-α, IL-17A, IL-6 and IL-13 compared to Nrp-1^-^CD8^+^ re-stimulated T cells (Fig 5D). Similar results were obtained after re-stimulation with lower concentrations of αCD3 and αCD28 (0.5 μg/ml or 0.1 μg/ml) (S4 Fig). Thus, we conclude that Nrp-1^+^CD8^+^ T cells are functional and highly activated.

### Ablation of Nrp-1 expression in T cells reduces the number of activated T cells in the brain of PbA-infected mice and ameliorates ECM outcome

**Fig 6:**
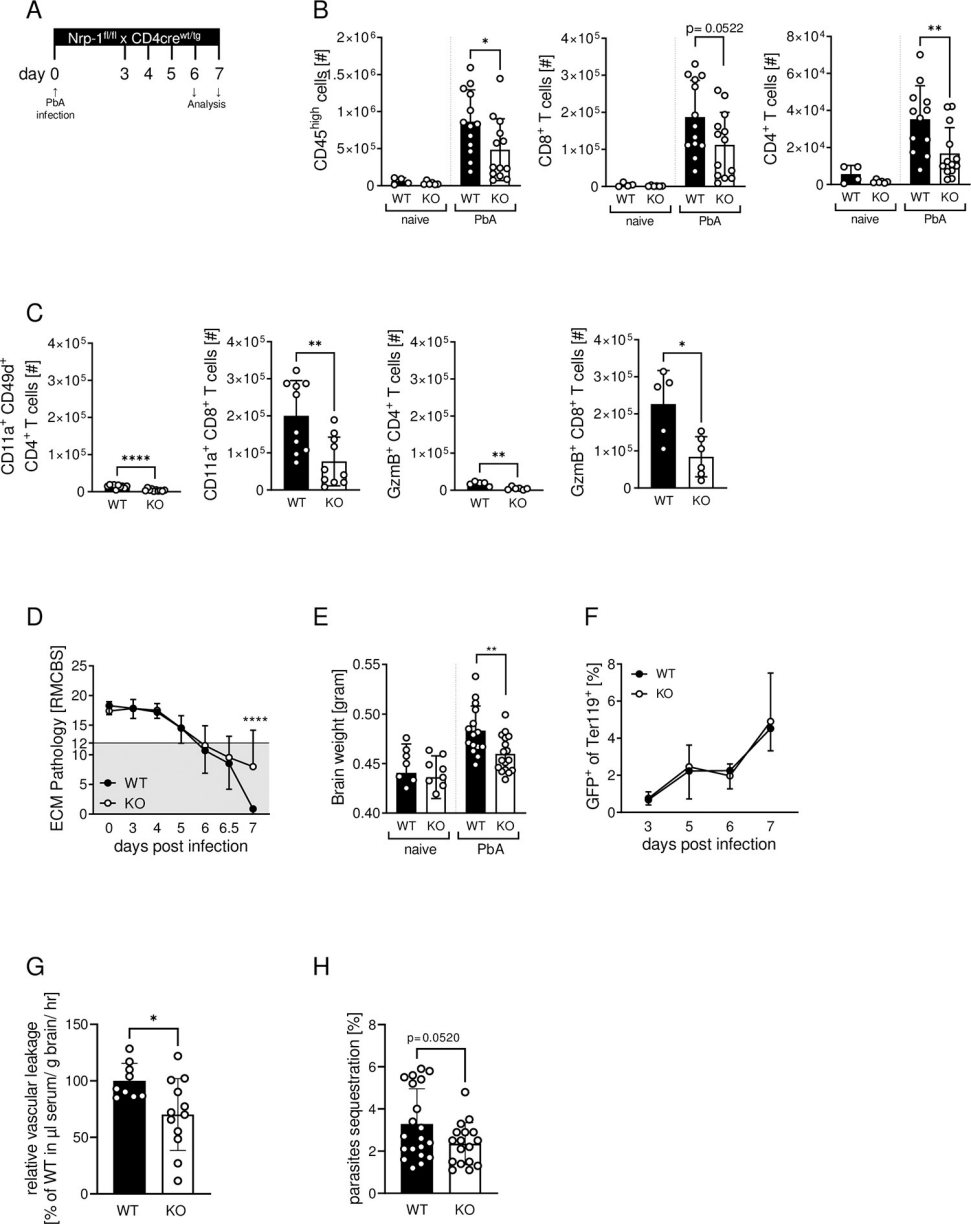
Ablation of Nrp-1 expression in T cells reduces the number of activated T cells in the brain of PbA-infected mice and ameliorates ECM outcome. (A) Nrp-1^fl/fl^ x CD4cre^tg^ (KO, white bars) and Nrp-1^fl/fl^ x CD4cre^wt^ littermates (WT, black bars) were infected i.v. with 10^5^ PbA-infected red blood cells (iRBCs) at day 0. The severity of ECM was assessed by the RMCBS score and immune cells were analyzed on day 6 or 7, when PbA-infected mice had a RMCBS score below 12. (B) The number of CD45^high^ peripheral immune cells, particularly CD4^+^ and CD8^+^ T cells, and (from left to right in C) the number of antigen-experienced and GzmB^+^ CD4^+^ and CD8^+^ T cells were quantified in the brain of naïve mice or at 6 or 7 days after PbA infection by flow cytometry. (D) ECM pathology was assessed by RMCBS score during the course of infection. Mice with a score below 12 were considered to have ECM. (E) Brain weight was assessed after cardiac perfusion on day 6 or 7 post infection. (F) Parasitemia was determined by flow cytometry and calculated as the proportion of GFP^+^ PbA-infected RBCs to total Ter119^+^ erythrocytes on day 3, 5, 6 and 7 after PbA infection. (G) Blood-brain barrier leakage was determined by injecting Evans Blue i.v. into PbA-infected Nrp-1WT and Nrp-1KO mice and analyzing Evans Blue concentration in serum and blood 2h later. (H) Parasite sequestration of GFP^+^ PbA into the brain was determined by flow cytometry. (B) Data from two to four independent experiments with n = 4–15 mice in total, (C) two to three independent experiments with n = 5–10 mice in total, (D and F) five independent experiments with n = 23–24 mice in total, (E) three to four independent experiments with n = 8–17 mice in total, (G) three independent experiments with n = 9–12 in total and (H) four independent experiments with n = 17–20 mice in total are presented as mean values with SD. Statistical significance was calculated with unpaired Student’s t test and for ECM pathology and parasitemia (D, E) with ordinary 2-way ANOVA and Sidak’s multiple comparisons test. *, p<0.05; **, p<0.01.

To further investigate the functional impact of Nrp-1 expression on T cells during an ongoing immune response *in vivo*, we infected T cell-specific Nrp-1-deficient mice with PbA and analyzed the T cell phenotype and ECM progression (Fig 6A). In these previously characterized Nrp-1^fl/fl^ x CD4cre^tg^ mice (Nrp-1KO) [5], exon 2 of the *nrp-1* gene is flanked by loxP sites [37]. Due to the activity of cre recombinase in CD4^+^ as well as CD4^+^CD8^+^ double-positive T cells during development in the thymus, Nrp-1KO mice have a CD4^+^ and CD8^+^ T cell-specific Nrp-1 ablation (S5A–S5C Fig). In naïve mice, Nrp-1 deficiency has no effect on the frequencies of CD4^+^ and CD8^+^ T cells in the spleen (S5D Fig).

During PbA infection, T cell-specific deletion of Nrp-1 resulted in significantly reduced numbers of CD45^high^ peripheral immune cells in the brain of Nrp-1KO mice compared to Nrp-1^fl/fl^ x CD4cre^wt^ (Nrp-1WT) littermates (Fig 6B). Specifically, the absolute number of CD4^+^ and CD8^+^ T cells was decreased. In contrast, we did not detect any significant difference in absolute numbers of CD8^+^ T cells in the spleen and blood of PbA-infected Nrp-1KO and Nrp-1WT mice (S6A and S6B Fig). Previous work suggested that, not only the number, but also the activation status of T cells is crucial for the development of neurological symptoms during cerebral malaria [38]. Indeed, flow cytometry analysis showed that absent Nrp-1 expression on T cells was associated with significantly lower numbers of antigen-experienced CD11a^+^CD49d^+^CD4^+^ T cells and CD11a^+^CD8^+^ T cells in the brain of Nrp-1KO mice in comparison to Nrp-1WT littermates (Fig 6C, left). In addition, we found less cytotoxic GzmB^+^CD4^+^ and GzmB^+^CD8^+^ T cells in the brain of PbA-infected Nrp-1KO mice than in Nrp-1WT mice (Fig 6C, right), whereas the absolute numbers of CD11a^+^CD8^+^ and GzmB^+^CD8^+^ T cells in the spleen and blood were similar between infected Nrp-1KO mice and Nrp-1WT mice (S6A and S6B Fig). Interestingly, we obtained similar results from *in vitro* stimulation experiments. MACS-sorted and αCD3/αCD28 (1 μg/ml, 0.5 μg/ml or 0.1 μg/ml) stimulated CD8^+^ T cells from the spleen of naïve Nrp-1KO mice and Nrp-1WT mice showed no differences in the expression of CD69, CD44, PD-1, GzmB or Ki-67 (S7 Fig). Nevertheless, ablation of Nrp-1 in CD4^+^ and CD8^+^ T cells attenuated the severity of ECM, as evidenced by a higher RMCBS score of Nrp-1KO mice compared to Nrp-1WT mice suffering from severe ECM (Fig 6D). Brain weight, an initial indicator for brain edema and neuroinflammation, was significantly reduced upon deletion of Nrp-1 expression on T cells during PbA infection, whereas blood parasitemia was comparable between the groups (Fig 6E and 6F). Since we did not detect any differences in the activation state of CD8^+^ T cells from Nrp-1KO mice and Nrp-1WT mice in the spleen or blood after PbA infection or upon stimulation *in vitro*, Nrp-1 seems to be a marker for activated CD8^+^ T cells, but dispensable for efficient initial T cell priming. In addition to the initial priming of CD8^+^ T cells, another important feature during ECM development is the cytotoxicity of CD8^+^ T cells resulting in the disruption of the blood-brain barrier (BBB) and immune cell infiltration [23]. Therefore, we analyzed the BBB leakage of PbA-infected Nrp-1KO and Nrp-1WT mice by i.v. injection of Evans Blue, followed by analysis of its distribution in brain and serum. In accordance with reduced absolute numbers of T cells in the brain (Fig 6B), T cell-specific Nrp-1 deficient mice showed significantly less vascular leakage after PbA infection than Nrp-1WT mice (Fig 6G). Moreover, we detected less parasite sequestration in the brain of infected Nrp-1KO mice compared to Nrp-1WT littermates (Fig 6H), which has been associated with severe disease [39].

To finally examine whether the effects observed in Nrp-1KO mice were indeed caused by ablation of Nrp-1 expression in effector T cells but not in Tregs, we infected Nrp-1^fl/fl^ x Foxp3cre^tg^ mice characterized by Treg-specific ablation of Nrp-1. PbA infection did not result in altered numbers of peripheral immune cells in the brain compared to infected WT littermates (S8A Fig). Neither the severity of ECM, nor brain weight of PbA-infected mice were affected by Nrp-1 depletion in Tregs (S8B and S8C Fig). Thus, the improvement of ECM in Nrp-1KO mice can be more likely attributed to ablation of Nrp-1 expression on conventional CD4^+^ and CD8^+^ T cells rather than on Tregs.

### CD8^+^ T cells upregulate Nrp-1 expression during LCMV infection and T cell-specific Nrp-1 ablation ameliorates immunopathology

**Fig 7:**
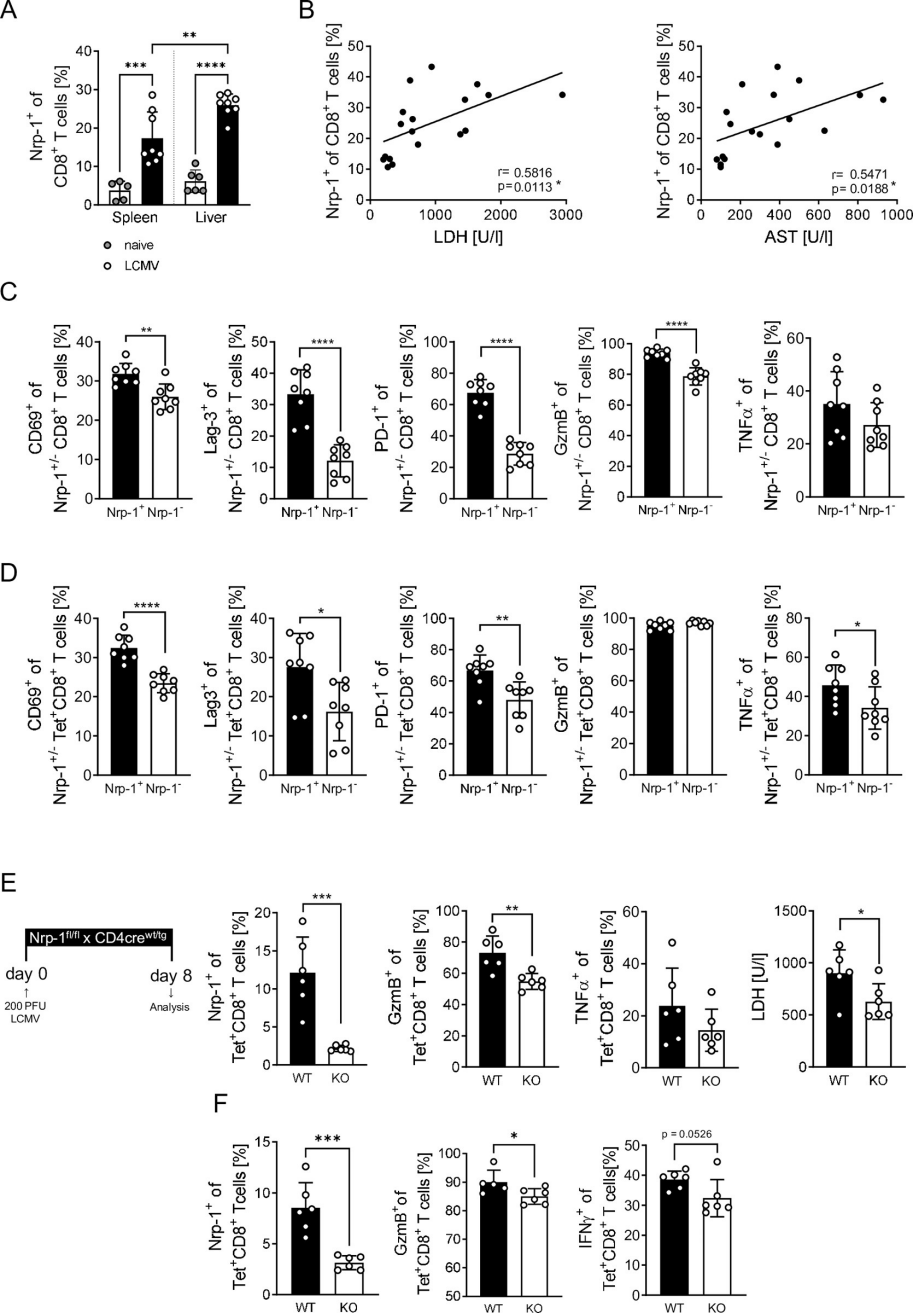
CD8^+^ T cells upregulate Nrp-1 expression during LCMV infection and T cell-specific Nrp-1 ablation ameliorates immunopathology. C57BL/6 mice (A-D) or Nrp-1^fl/fl^ x CD4cre^tg^ (KO, white bars) and Nrp-1^fl/fl^ x CD4cre^wt^ littermates (WT, black bars) (E, F) were infected with 200 PFU LCMV-WE i.v. and T cell phenotypes were analyzed in the spleen 8 days post infection. (A) Nrp-1 expression on CD8^+^ T cells was measured by flow cytometry in the spleen and liver of naïve (white bar, grey dots) and LCMV-infected C57BL/6 mice (black bars, white dots). (B) The correlation of the frequency of Nrp-1^+^CD8^+^ T cells in the spleen with serum LDH and AST concentrations was determined in C57BL/6 mice infected with 200 PFU LCMV-WE, 200 PFU LCMV-docile and 2x10^6^ PFU LCMV-docile on day 8 post infection. (C and D) Expression of CD69, Lag-3, PD-1, GzmB and TNF-α was determined on Nrp-1^+^ (black bars) and Nrp-1^-^ CD8^+^ T cells (white bars) (C) or Tet^+^CD8^+^T cells (D). (E and F) Nrp-1, GzmB, TNF-α or IFN-γ expression was determined on antigen-specific Tet^+^CD8^+^ T cells in the spleen (E) and in the liver (F) of infected Nrp-1WT or Nrp-1KO mice by flow cytometry. LDH concentrations were measured in the serum at day 8 post LCMV infection. Data from two independent experiments with n = 5–8 mice per group are shown as mean values with SD. Statistical significance was calculated with (A) ordinary one-way ANOVA and Tukey’s multiple comparisons test. (B) Correlation is based on two independent experiments with n = 18 mice and statistics were analyzed using Pearson correlation coefficients. P and r values are displayed in the graphs. (C-F) Significance was tested using unpaired Student’s t test. *, p<0.05; **, p<0.01; ***, p<0.001; ****, p<0.0001.

As CD8^+^ effector T cells do not only play an important role in the development of cerebral malaria, but also in other infectious diseases, we hypothesized that Nrp-1 induction might be a more general mechanism. Therefore, we analyzed Nrp-1 expression on CD8^+^ T cells during an acute LCMV-WE infection, in which the cytotoxic function of CD8^+^ T cells is crucial for virus clearance but also responsible for emerging immunopathology [40]. On day 8 of acute LCMV infection, 17% of CD8^+^ T cells in the spleen and 26% in the liver of infected C57BL/6 mice expressed Nrp-1 on the surface (Fig 7A). In line with our results from ECM, the percentage of CD8^+^ T cells expressing Nrp-1 on the surface correlated with serum lactate dehydrogenase (LDH) and aspartate transaminase (AST) levels associated with liver damage during LCMV infection (Fig 7B). Flow cytometry analyses showed the same activated CD8^+^ T cell phenotype within the Nrp-1^+^CD8^+^ T cell subset as observed in mice with cerebral malaria. Nrp-1^+^CD8^+^ T cells from LCMV-infected mice expressed significantly higher levels of CD69, Lag-3, PD-1, GzmB and TNF-α than Nrp-1^-^CD8^+^ T cells (Fig 7C). This highly activated phenotype was also present in antigen-specific (Tet^+^) Nrp-1^+^ compared to Nrp-1^-^ CD8^+^ T cells (Fig 7D).

Interestingly, LCMV infection of T cell-specific Nrp-1-deficient mice (Nrp-1KO) resulted in reduced percentages of cytotoxic (GzmB^+^) and pro-inflammatory cytokine (TNF-α or IFN-γ) producing CD8^+^Tet^+^ T cells in the spleen and the liver compared to wild-type littermates (Fig 7E and 7F). Moreover, serum LDH concentration was decreased suggesting milder liver damage (Fig 7E). Hence, induction of Nrp-1 expression on CD8^+^ T cells appears to be a general mechanism in acute parasitic and viral infections associated with exacerbated disease severity.

## Discussion

We have previously described that stimulation-induced Nrp-1 expression on CD4^+^ effector T cells is associated with strong T cell activation that promotes early disease in a diabetes mouse model [13]. In cancer, other groups have shown that Nrp-1 expression on CD8^+^ T cells is induced during disease progression [15, 18]. However, the function of Nrp-1 on CD8^+^ T cells in acute parasitic and viral infections remains unclear. Here, we demonstrated that cerebral malaria and acute LCMV infection led to induction of Nrp-1 on CD8^+^ T cells, which is accompanied by a highly activated T cell phenotype and correlates with disease severity and tissue damage. Strikingly, T cell-specific ablation of Nrp-1 ameliorated disease severity of ECM and LCMV infection-induced liver pathology.

With the onset of the first neurological symptoms of ECM on day 5 post PbA infection (S1A Fig), we measured a significant increase of Nrp-1 expression in CD8^+^ T cells in the spleen, blood and brain (Fig 1). This correlated with the time point at which we observed highly significant activation of CD8^+^ T cells in the spleen (S1C Fig). Shortly after, at day 6, a strong infiltration of immune cells into the brain was detected, which is likely the time of disruption of the blood-brain barrier caused by cytotoxic CD8^+^ T cells [23]. Interestingly, the frequency of total CD8^+^ T cells in the brain of PbA-infected C57BL/6 mice was independent of the severity of ECM as quantified by the RMCBS score (Fig 1F, left). This observation is consistent with findings in human *P*. *falciparum* infection that demonstrated higher severity of malaria disease associated with increased frequencies of cytotoxic GzmB^+^ and activated CD38^+^ or PD-1^+^CD8^+^ T cells, while the absolute number of CD8^+^ T cells was independent of disease severity [38]. In mice, it was also shown that GzmB expression is essential for the development of ECM [24]. Thus, the activation state and effector function of CD8^+^ T cells are critical for disease progression.

Microarray and flow cytometric analyses revealed that Nrp-1^+^CD8^+^ T cells from PbA- and LCMV-infected mice are highly activated in comparison to Nrp-1^-^CD8^+^ T cells (Figs 2, 3 and 7). Nrp-1^+^CD8^+^ T cells showed enhanced expression of molecules involved in T cell effector functions such as CD69, GzmB and IFN-γ (Fig 3). Moreover, we found that Nrp-1^+^CD8^+^ T cells showed elevated expression of molecules associated with an inhibitory phenotype (PD-1, CTLA-4, Tim-3 and Lag-3) compared to Nrp-1^-^CD8^+^ T cells. The same phenotype has been observed in cancer: Nrp-1 expression on CD8^+^ T cells was accompanied by elevated expression of CD44, CD69 and CD25, but also by several co-inhibitory molecules such as PD-1, CTLA-4, Lag-3 and Tim-3 [15, 18]. Therefore, the authors suggested that Nrp-1 is an inhibitory receptor of CD8^+^ T cells. In PbA infection, T cells are described to frequently express exhaustion-associated molecules, but many studies imply that this is not due to exhaustion of these cells in general but rather due to their activation instead. For example, a previous study showed that Lag-3^+^ T cells were more cytotoxic (GzmB^+^) and produced higher levels of IFN-γ than Lag-3^-^ T cells [41]. Furthermore, analysis of a subset of CD160^+^CD8^+^ T cells identified a comparable expression pattern to Nrp-1^+^CD8^+^ T cells; CD160^+^CD8^+^ T cells expressed increased PD-1, Klrg-1, Ki-67 and GzmB compared to CD160^-^CD8^+^ T cells and their frequency correlated with the severity of ECM [42]. An *in vitro* cytotoxicity assay as well as the measurement of cytokine release of CD160^+^ or CD160^-^ CD8^+^ T cells confirmed the activation and functionality of CD160^+^CD8^+^ T cells rather than their exhaustion. In accordance, we demonstrated that *in vitro* re-stimulation of Nrp-1^+^CD8^+^ T cells stabilized the strong activation phenotype of these cells and cytokine production was significantly enhanced compared to Nrp-1^-^CD8^+^ re-stimulated T cells (Fig 5). Therefore, our results provide evidence that Nrp-1^+^CD8^+^ T cells are strongly activated.

T cell-specific ablation of Nrp-1 expression in Nrp-1KO mice resulted in less severe neurological deficits and improved ECM outcome compared to Nrp-1WT littermates (Fig 6D). According to our cell-specific analyses, Nrp-1 deficient T cells seem to be less activated, which is supported by reduced numbers of antigen-specific CD11a^+^ and cytotoxic GzmB^+^ T cells in the brain of PbA-infected Nrp-1KO (Fig 6C). We are aware of the fact that in the transgenic mouse model used in the present study, Nrp-1^fl/fl^ x CD4cre^tg^ mice also lack Nrp-1 expression on conventional CD4^+^ T cells and Tregs, which are known to express Nrp-1 on their surface (S2, S5A and S5B Figs). This is an issue that arises due to the CD4^+^CD8^+^ double-positive T cell stage during development in the thymus and is not easy to remedy. However, by PbA infection of Treg-specific Nrp-1 deficient mice (Nrp-1^fl/fl^ x Foxp3cre^tg^) we could exclude an effect of Treg-derived Nrp-1 on the immune cell infiltration in the brain and ECM development (S8 Fig). In general, the number of cerebral Tregs is very low during ECM and targeted depletion of Tregs with diphtheria toxin in DEREG mice led to negligible changes in numbers of activated T cells in the brain and ECM manifestation [43]. Therefore, Tregs seem to play a minor role in regulating the immune response during ECM and, thus, Nrp-1 expression by Tregs seems to be negligible in the Nrp-1KO model. Additionally, we previously showed that induction of Nrp-1 expression on effector CD4^+^Foxp3^-^ T cells is associated with a high activation state of these cells [13]. We cannot exclude that this Nrp-1 expression on effector CD4^+^ T cells has an impact on manifestation of ECM and LCMV in Nrp-1KO mice. However, in the brain of PbA-infected mice, we found a much higher number of Nrp-1-expressing CD8^+^ T cells compared to CD4^+^ T cells (Figs 1D and S2C). In addition, it is well established that pathogenic CD8^+^ T cells play a central role in the development of ECM and liver injury in LCMV infections [23, 40]. Therefore, we hypothesize that Nrp-1 expression on CD8^+^ T cells rather than on CD4^+^ T cells is the key player in ECM and LCMV, which needs to be verified in further studies.

*In vitro*, we found that Nrp-1 is rapidly induced on CD8^+^ T cells after stimulation with αCD3 and αCD28, but expression is only transient and decreases over time (Fig 4C). By re-stimulation with αCD3 and αCD28, we demonstrated that stable Nrp-1 expression requires a continuous TCR stimulus. This finding is supported by a recent study in which repeated stimulation of CD8^+^ T cells with αCD3/αCD28 beads was used to mimic chronic TCR stimulation, resulting in stable Nrp-1 expression on CD8^+^ T cells after three cycles of stimulation [18]. Therefore, Nrp-1 induction seems to be a general effect of TCR stimulation and Nrp-1 may affect the activation phenotype of CD8^+^ T cells associated with cytotoxic GzmB release and production of pro-inflammatory cytokines. This hypothesis is supported by the fact that we were able to show similar results in acute LCMV infection (Fig 7). Here, the frequency of Nrp-1^+^CD8^+^ T cells, representing activated cells, consistently correlated with the extent of immune-induced liver damage. *In vitro*, we did not detect any differences in the expression of activation-associated molecules such as CD69, CD44, and Ki-67 between CD8^+^ T cells from Nrp-1KO mice and CD8^+^ T cells from Nrp-1WT mice upon a single stimulation with αCD3/αCD28 (S7 Fig). This could imply that Nrp-1 may not affect initial T cell priming, but one has to keep in mind that naïve CD8^+^ T cells from Nrp-1WT mice also lack Nrp-1 expression. Hence, one would expect comparable responses to primary stimulation from both Nrp-1 deficient and Nrp-1 proficient CD8^+^ T cells. Nevertheless, our results provide evidence for reduced cytotoxic function of Nrp-1-deficient CD8^+^ T cells exacerbating immunopathologies induced by both, acute PbA infection and LCMV infection. During PbA infection the BBB is disrupted due to the recognition of MHC-I presented *Plasmodium*-specific antigens on endothelial cells by cytotoxic CD8^+^ T cells. Since T cell-specific ablation of Nrp-1 in PbA-infected mice resulted in a significantly reduced vascular leakage (Fig 6G), our results indicate that Nrp-1 expression correlates with the cytotoxic activity of CD8^+^ T cells. However, we could not exclude that Nrp-1 also affects other features of CD8^+^ T cells that contribute to the pathology in infected mice. Nrp-1 has been described to be involved in the migration of several cell types including gastric cancer cells [44], glioma cells [45] and human endothelial cells [46] towards its ligand VEGF. We identified Nrp-1 to facilitate the migration of CD4^+^Foxp3^+^ Tregs into tumor tissues in response to VEGF [5]. Well in line, Gaddis and colleagues demonstrated that Nrp-1^+^CD4^+^Foxp3^-^ non-Tregs migrate more efficiently towards a VEGF gradient *in vitro* than Nrp-1^-^CD4^+^Foxp3^-^ non-Tregs. From these results and *in vivo* data, they concluded that Nrp-1 aids in the migration of CD4^+^ effector T cells into the aortic tissue and draining lymph nodes and worsen the outcome of atherosclerosis [14]. Thus, whether Nrp-1 might also have an impact on the migratory capacity of pathologic CD8^+^ T cells during parasitic and viral infections in addition to its effect on the cytotoxic activity has to be clarified in further studies.

Acute activation of T cells during ECM and LCMV infection might be one reason for the controversial results on the role of Nrp-1 in CD8^+^ T cells in our study compared to studies performed in tumor-bearing mice or mice infected with gamma-herpes virus [15, 17, 18]. The authors found no effect on primary tumor growth or persistent virus infection, and only reported an increased frequency of tumor-free mice with a CD8^+^ T cell-specific Nrp-1 ablation compared to wild-type mice when secondary tumors were re-transplanted or upon re-infection, respectively [17, 18]. Therefore, they focused on Nrp-1 expression in the context of memory formation. In both, the mouse tumor model and persistent gamma-herpes virus infection, the effect of Nrp-1 ablation was not analyzed in the early phase of disease, but approximately three weeks after inoculation or infection. In contrast, we performed our analysis in the acute phase of infection. Nevertheless, we detected reduced MPECs among Nrp-1^+^CD8^+^ T cells in PbA-infected mice (Fig 3D), which suggests that Nrp-1 might also interfere with effective T cell memory development during *Plasmodium* infection, providing a promising starting point for future studies.

Overall, we analyzed the impact of T cell-expressed Nrp-1 in two distinct infections models, in which overwhelming CD8^+^ T cell responses induce immunopathologies in the brain and the liver. In other viral and parasitic infections, cytotoxic CD8^+^ T cells might have a protective effect by efficiently clearing pathogens rather than causing harmful pathologies. Of particular interest will be the investigation of Nrp-1 on CD8^+^ T cell responses during these infectious diseases to understand the general impact of Nrp-1.

In conclusion, our data provide evidence that Nrp-1 was rapidly induced on CD8^+^ T cells during PbA infection, acute LCMV as well as *in vitro* stimulation in the presence of αCD3 and αCD28. Nrp-1^+^CD8^+^ T cells were highly activated and required a continuous stimulus for stable Nrp-1 expression. In murine cerebral malaria and acute LCMV infection, Nrp-1 expression correlated with disease severity and, strikingly, T cell-specific ablation of Nrp-1 improved the outcome of ECM and ameliorated immunopathology in LCMV-infected mice. Since the activation state of CD8^+^ T cells plays a critical role in many pathologies, Nrp-1 might serve as an early prognostic marker, and blocking of Nrp-1 expression could be a promising therapeutic target in various pathologies induced by exacerbated CD8^+^ T cell responses.

## Material and methods

### Ethics statement

All animal experiments were performed in accordance with the guidelines of the German Animal Welfare Act, and recommendations of the Federation of European Laboratory Animal Science Association (FELASA). The experiments were approved by the ethics committee of the State Authority for Nature, Environment and Customer Protection (LANUV) of North-Rhine-Westphalia, Germany (approval number: 81–02.04.2020.A404).

### Mice

C57BL/6J wild-type mice were purchased from Envigo or Charles River. Nrp-1^fl/fl^ mice were kindly provided by D.D. Ginty (John Hopkins University, Baltimore, MD; [37]) and crossed with CD4cre mice or Foxp3cre (FIC) mice, kindly provided by S. Sakaguchi [47]. Nrp-1tdTomato mice that co-express Nrp-1 and the tdTomato protein under the control of the Nrp-1 promoter were generated by Taconic Bioscience (Cologne, Germany) [13]. OT-I mice express a transgenic T cell receptor recognizing ovalbumin peptide 257–264 [48] were kindly provided by T. Yevsa, Hannover Medical School, Germany. All mice were kept in individually ventilated cages under specific pathogen-free conditions at the animal facility of the University Hospital Essen. All animal experiments were performed in accordance with the guidelines of the German Animal Welfare Act and are approved by the State Authority for Nature, Environment and Customer Protection (LANUV) of North-Rhine-Westphalia, Germany.

### *Plasmodium* and LCMV infection

For *Plasmodium* infection, red blood cells (RBCs) infected with *Plasmodium berghei* ANKA parasite strain PbGFPko230p-SM_CON_ (exp 507 clone 1) with a single *gfp* copy integrated into the 230p locus [49] were cryopreserved and passaged once into wild-type mice before use. Experimental mice were infected by intravenous (i.v.) injection of 10^5^ infected RBCs (iRBCs). Parasitemia was determined by quantification of GFP^+^iRBCs within the Ter119^+^-erythrocyte population by flow cytometry. Neurological deficits due to the manifestation of ECM were quantified blinded using the Rapid-Murine-Coma-and-Behavior-Scale (RMCBS) according to Carroll and colleagues [29]. This score includes 10 parameters assessing coordination, exploratory and hygienic behavior, strength and muscle tone as well as reflexes and self-preservation, each scored from 0 (lowest function) to 2 (highest function). Healthy mice usually have a score of 18–20, and as the disease progresses, the score decreases until animals with a score under 12 are considered to have cerebral malaria [29]. Mice were usually analyzed between day 6 and 7 post infection. To study viral infections, mice were infected by i.v. administration of 200 PFU LCMV-WE (and 200 or 2x10^6^ PFU LCMV-docile for correlation analysis) and animals were analyzed on day 8 post infection.

### Cell Isolation for flow cytometry analyzes

Brains were dissected after cardiac perfusion with 20 ml PBS and homogenized in 1% HEPES-buffered RPMI1640 through cell strainers. Peripheral immune cells were obtained from cerebral cell suspensions after density centrifugation for 20 minutes at 2800 x g in 37% Percoll with 0.01 M HCl/PBS. Blood samples were repeatedly incubated with red blood cell lysis buffer to remove erythrocytes from circulating immune cells. To obtain splenic single cell suspensions, spleens were passed through cell strainers together with red blood cell lysis buffer and PBS supplemented with 2% FCS and 2 mM EDTA. For the isolation of single cell suspensions from livers, livers were cut into pieces and digested with 240 μg Collagenase D and 30 μg DNase for 45 minutes at 37°C. Subsequently, tissue fragments were homogenized through cell strainers and treated with red blood cell lysis buffer before further processing.

### *In vitro* activation, Nrp-1 stability and re-stimulation

For *in vitro* assays, CD8^+^ T cells were enriched from splenic single cell suspensions by magnetic-activated cell sorting (MACS) using the CD8^+^ T cell isolation kit from Miltenyi Biotec (Bergisch Gladbach, Germany) according to the manufacturer’s instructions. Purified CD8^+^ T cells were stimulated with 0.1 μg/ml, 0.5 μg/ml or 1 μg/ml plate-bound αCD3/soluble αCD28 at 37°C. Alternatively, CD8^+^ T cells were isolated from OT-I mice and stimulated with 0.1 μg/ml, 0.5 μg/ml or 1 μg/ml OVA-peptide in the presence of irradiated splenocytes from WT mice as antigen-presenting cells. The activation phenotype of Nrp-1^+^ and Nrp-1^-^ CD8^+^ T cells was analyzed by flow cytometry after 48 hours of activation. To study the stability of Nrp-1 expression, we sorted Nrp-1^+^CD8^+^ T cells by FACS after 48 hours of *in vitro* stimulation. Cells were re-cultivated in the presence of 250 U/ml IL-2 and Nrp-1 expression was measured by flow cytometry after 24 to 96 hours of re-cultivation. To test the functionality of Nrp-1^+^ and Nrp-1^-^ CD8^+^ T cells, we used MACS-purified CD8^+^ T cells from Nrp-1tdTomato reporter mice or C57BL/6 mice and stimulated them as described above. We sorted Nrp-1tdTomato^+^CD8^+^ T cells or Nrp-1^+^CD8^+^ T cells after 48 hours of activation and re-stimulated them again for 48 hours in the presence of 0.1 μg/ml, 0.5 μg/ml or 1 μg/ml plate-bound αCD3/soluble αCD28.

### Quantification of cytokine concentrations with Luminex Technology

To quantify cytokine concentrations in the supernatant of re-stimulated Nrp-1^+^ and Nrp-1^-^ CD8^+^ T cells, samples were processed using polystyrene bead-based Luminex technology (R&D Systems, Minneapolis, MN) according to the manufacturer’s protocol. Measurements were performed with the MAGPIX Luminex instrument; and data were analyzed with the Luminex xPONENT software (Luminex Corporation, Austin, TX).

### Quantification of liver enzyme activity

The activity of lactate dehydrogenase (LDH) and aspartate transaminase (AST) was quantified in the serum of LCMV-WE and LCMV-docile-infected mice in the Central Laboratory of the University Hospital in Essen, Germany.

### Evans Blue

For studying blood-brain barrier (BBB) leakage, 50 μl 3% Evans Blue (AppliChem, Darmstadt, Germany) in PBS was injected i.v. in PbA-infected mice at day 5. After 2h, serum samples were taken. Brains were dissected after cardiac perfusion with 20 ml cold PBS, weighted, put into 750 μl formamide (Sigma-Aldrich, Munich, Germany) and incubated for 24h at 37°C for Evans Blue extraction. Formamide was aspirated and Evans Blue absorbance for serum and brain samples was determined at 620 nm using an EnVision 2104 multilabel reader. BBB leakage was calculated as Evans Blue concentration brain x extraction volume (750 μl))/Evans Blue concentration serum/brain mass/hours of circulation (2h), yielding μl serum/g brain/hr.

### Antibodies, tetramer staining and flow cytometry

The following fluorochrome-conjugated antibodies were used for flow cytometry analyses: αCD45, αCD11a, αPD-1, αCD160, αTer119 (all from BioLegend, San Diego, CA), αCD4, αCD49d, αLag-3, αCD44, αCTLA-4, αIFN-γ (BD Biosciences, Heidelberg, Germany), αKlrg-1, αCD69, αKi-67, αFoxp3, αTNF-α (eBioscience; Thermo Fisher Scientific, Langenselbold, Germany), αTim-3 (R&D Systems, Minneapolis, MN), αGzmB (Invitrogen, Karlsruhe, Germany), αCD8 (BD Bioscience, Heidelberg, Germany/eBioscience; Thermo Fisher Scientific, Langenselbold, Germany) and αNrp-1 (R&D Systems, Minneapolis, MN/eBioscience, Thermo Fisher Scientific, Langenselbold, Germany). Dead cells were identified by staining with Fixable Viability Dye eFluor 780 (eBioscience; Thermo Fisher Scientific, Langenselbold, Germany). For intracellular staining, cells were treated with Foxp3 staining kit (eBioscience, Thermo Fisher Scientific, Langenselbold, Germany) according to the manufacturer’s protocol. Cytokine expression was measured in splenocytes re-stimulated with 100 ng/ml phorbol 12-myristate 13-acetate (PMA) and 1 μg/ml ionomycin in the presence of 5 μg/ml brefeldin A (all three by Sigma-Aldrich, Munich, Germany) for 4 hours at 37°C. After fixation with 2% paraformaldehyde and permeabilization with 0.1% IGEPAL CA-63 (Sigma-Aldrich, Munich, Germany), cells were stained with intracellular antibodies.

LCMV-specific CD8^+^ T cells were identified by staining with GP_33-41_ (KAVYNFATM) peptide-major histocompatibility complex class I tetramer (GP-33-41/H-2D^b^) (NIH tetramer core facility, Atlanta, GA) that was labelled with allophycocyanin (APC) for 15 minutes at 4°C, followed by antibody staining as described before.

In general, flow cytometric analysis were performed on gated lymphocytes (SSC/FSC-A), singlets (FSC-A/FSC-H) and CD8^+^ T cells or CD4^+^ T cells with exclusion of dead cells by FvD staining similar as shown in Fig 1A. In some experiments CD8^+^ T cells were additionally separated into Nrp-1^+^ and Nrp-1^-^ CD8^+^ T cells (Figs 3, 5B, 5C and 7C) or Nrp-1^+^ and Nrp-1^-^ CD8^+^Tet^+^ T cells and analyzed for the expression of indicated molecules (Fig 7D and 7E). For analyzing brain samples, living single cell lymphocytes were additionally gated on CD45^high^ cells prior to further analysis of CD8^+^ or CD4^+^ T cells.

For determination of GFP^+^ PbA sequestration into the brain by flow cytometry, cell debris was excluded (SSC/FSC-A) from total brain cells, followed by gating on singlets (FSC-A/FSC-H) and analysis of the frequency of GFP^+^ parasites.

Flow cytometry measurements and cell sorting were performed with a LSR II instrument (BD Biosciences, Heidelberg, Germany) and FACS Aria II (BD Biosciences, Heidelberg, Germany), respectively. Acquisition and analyses were performed with FACS DIVA (BD Biosciences, Heidelberg) or FlowJo.

### Microarray

For microarray analysis, CD8^+^ T cells were isolated from spleens of PbA-infected C57BL/6 mice via FACS as described in the previous sections. 2,000 Nrp-1^+^ and Nrp-1^-^ CD8^+^ T cells were sorted directly into single cell lysis buffer of the Invitrogen single cell lysis kit (Thermo Fisher Scientific, Waltham, USA) and samples were further processed according to the manufacturer’s protocol. RNA was isolated with the Qiagen RNAeasy kit (Qiagen, Hilden, Germany) and approximately 2 ng of RNA were labelled with biotin using the GeneChip Pico Kit (Affymetrix, Santa Clara, CA) as recommended by the manufacturer. 5.5 μg of biotinylated cDNA were fragmented and hybridized to an identical lot of Affymetrix Clarion S (400 Format) in a hybridization cocktail with BioB, BioC, BioD and Cre as hybridization controls, for 17 hours at 45°C. Hybridization was performed for 16 hours as described by the manufacturer, before Clariom S chips were washed and stained in an Affymetrix Fluidics Station 450. GeneChips were scanned with the Affymetrix GCS 3000 and analysis of images was performed with Affymetrix GeneChip Command Console Software (AGCC) and Affymetrix Expression Console Software (Affymetrix, Santa Clara, CA). Data was analyzed as previously described [50].

Data has been stored in NCBI’s Gene Expression Omnibus and is accessible with GEO series accession number GSE199569.

### Realtime RT-PCR

RNA was isolated from FACS-sorted Nrp-1^+^ and Nrp-1^-^ CD8^+^ T cells from PbA-infected C57BL/6 mice using the RNeasy Mini Kit (Qiagen, Hilden, Germany) according to the manufacturer’s instructions. 100 ng of RNA was reversed transcribed using M-MLV Reverse Transcriptase (Promega, Waldorf, Germany) with dNTPs, Oligo-dT mixed with Random Hexamer primers (Thermo Fisher Scientific, Langenselbold, Germany). For quantitative real-time PCR Fast SYBR Green Master Mix (Thermo Fisher Scientific, Langenselbold, Germany) was used on a 7500 Fast Real-Time PCR System (Thermo Fisher Scientific, Langenselbold, Germany) with specific primers for Bcl2 (5`- CAG AGG GGC TAC GAG TGG GAT GCT—3`, 5`- CCG GCG GAG GGT CAG ATG GA—3`), CCL1 (5`- CTG CTG GCT GCC GTG TGG ATA C—3`, 5`- GGC GCA GCT TTC TCT ACC TTT GTT—3`), CD62L (5`- GAC ATG GGT GGG AAC CAA CA—3`, 5`- TGG ACC ACT GTG TAG CAG AGA—3`), IL7R (5`- CTC CCC CGT AGC TCA CAG AAG—3`, 5`- CCA ACA ACA GGG AAA ACA GAT T—3`), Tnfrsf4 (5`- AGG GCC CTG CAT TTG CTG TTC TC—3`, 5`- GTT GTC CGT GCC CCA TAA AAT CCA—3`), and RPS9 (5`- CTG GAC GAG GGC AAG ATG AAG C—3`, 5`- TGA CGT TGG CGG ATG AGC ACA—3`). Each sample was measured as technical duplicate. The expression levels of target genes were normalized against ribosomal protein S9 (RPS9).

### Statistics

All graphs (except radar plots) and statistics were performed with GraphPad Prism 9 (GraphPad Software, La Jolla, USA). Radar plots were generated using OriginPro2019b software (OriginLab, Northampton, USA). Data was tested for normality using D’Agostino & Pearson omnibus or Shapiro-Wilk normality test. In case of normality Student`s t-test for two samples or one-way ANOVA for multiple samples were used to calculate statistical significant differences. If data sets did not pass normality, non-parametric Mann-Whitney test (two samples) or Kruskal-Wallis test for multiple samples were used. The respective test used to calculate statistically significant differences is described in the figure legends. In general, biological replicates (one mouse or cells from one mouse) with mean ± SD are depicted as one dot. In case of technical duplicates or replicates (Figs 2C, 4B and 5C) the mean was included as single sample into the graphs and stated in the figure legend. In all analyses, p<0.05 was considered as statistically significant.

## Supporting information

S1 Fig *Plasmodium berghei ANKA* infection is associated with neurological deficits and activation of T cells. C57BL/6 mice were infected i.v. with 10^5^ *Plasmodium berghei ANKA* (PbA) GFP^+^-infected erythrocytes. (A) The severity of experimental cerebral malaria (ECM) was assessed by the RMCBS score, which quantifies neurological deficits during development of ECM. Mice with a RMCBS score below 12 are considered to have ECM. (B) Parasitemia was determined by flow cytometry and calculated as the proportion of GFP^+^PbA-infected RBCs of total Ter119^+^ erythrocytes on day 3, 5 and 6 after PbA infection. (C) The frequency of PD-1-, GzmB- and IFN-γ-expressing CD8^+^ T cells was measured by flow cytometry in the spleen. Results from (A, B) n = 5 mice (d0, 3, 4, 5) from one experiment, n = 11 mice in total (d6) from two experiments, (C) n = 3–5 mice (d3, 4, 5, 6) from one experiment and n = 9–11 mice (d0) from three experiments are shown as mean + SD. Each dot represents one animal. Statistical significance was calculated with ordinary one-way ANOVA and Dunett`s multiple comparisons test. ***, p<0.001; ****, p<0.0001.
(TIF)

S2 Fig *Plasmodium berghei ANKA* infection induces Nrp-1 expression on conventional CD4^+^ T cells and reduces frequencies of Nrp-1^+^Foxp3^+^CD4^+^ Tregs. (A and B) Nrp-1 expression on CD4^+^Foxp3^-^ conventional T cells and CD4^+^Foxp3^+^ Tregs was analyzed by flow cytometry on day 0 and in PbA-infected C57BL/6 mice at day 3, 4, 5 and 6 post infection in spleen and blood. (C) Frequencies and absolute numbers of peripheral CD4^+^ T cells of CD45^high^ cells were measured in the brain by flow cytometry. Results from 1–2 independent experiments with n = 5–11 mice per time point are shown as mean + SD. Each dot represents one animal. Statistical significance was calculated with nonparametric Kruskal-Wallis test with Dunn’s multiple comparisons test. *, p<0.05; **, p<0.01; ****, p<0.0001.
(TIF)

S3 Fig Antigen-specific Nrp-1^+^CD8^+^ T cells exhibit a more activated phenotype than Nrp-1^-^CD8^+^ counterparts. MACS-sorted CD8^+^ T cells isolated from spleen of OT-I mice were stimulated with indicated concentration of OVA in the presence of irradiated splenocytes as APCs for 48h. The expression of (A) Nrp-1 and (B) CD69, PD1 and GzmB on gated Nrp-1^+^CD8^+^ T cells and Nrp-1^-^CD8^+^ T cells was analyzed by flow cytometry. Results from three independent experiments with cells from n = 6–8 mice in total are summarized as mean ± SD. Statistical significance was calculated with one-way ANOVA and Tukey`s multiple comparisons test. **, p<0.01; ***, p<0.001; ****, p<0.0001.
(TIF)

S4 Fig Cytokine secretion of sorted Nrp-1^+^CD8^+^ and Nrp-1^-^CD8^+^ T cells re-stimulated with low concentrations of αCD3/αCD28. MACS-sorted CD8^+^ T cells from spleen of C57BL/6 mice were stimulated *in vitro* with 1 μg/ml αCD3/αCD28 for 48h. Nrp-1^+^CD8^+^ and Nrp-1^-^CD8^+^ T cells were sorted by FACS and re-stimulated with 0.5 μg/ml or 0.1 μg/ml αCD3 plate-bound/αCD28 soluble for another 48h. The concentration of cytokines in the supernatant was determined by Luminex technology. Data from 2 independent experiments with n = 3 mice per experiment are summarized as mean ± SD. Statistical analysis was performed with one-way ANOVA. *, p<0.05; **, p<0.01; ****, p<0.0001.
(TIF)

S5 Fig T cell-specific ablation of Nrp-1 expression in Nrp-1KO mice. Splenocytes from Nrp-1^fl/fl^ x CD4cre^wt^ (WT, black bars) and Nrp-1^fl/fl^ x CD4cre^tg^ (KO, white bars) littermates were cultured *in vitro* (unstimulated) and stimulated with αCD3 and αCD28 (stimulated) for 48 hours. Successful ablation of Nrp-1 expression in (A) Foxp3^-^CD4^+^ conventional T cells, (B) Foxp3^+^CD4^+^ Tregs and (C) CD8^+^ T cells was verified by flow cytometry and is shown as mean ± SD. Representative FACS plots including FMO controls are shown in the upper panels. Data from one experiment with n = 2 mice is depicted. (D) The percentages of CD8^+^ and CD4^+^ T cells in spleen of naïve Nrp-1^fl/fl^ x CD4cre^wt^ (WT) and Nrp-1^fl/fl^ x CD4cre^tg^ (KO) littermates were determined by flow cytometry. Data from 2–3 independent experiments with n = 9–12 mice in total are shown as mean ± SD. Each dot represents one animal.
(TIF)

S6 Fig T cell numbers and T cell activation in spleen and blood are not altered in Nrp-1KO mice upon PbA infection. Nrp-1^fl/fl^ x CD4cre^wt^ (WT, black bars) and Nrp-1^fl/fl^ x CD4cre^tg^ (KO, white bars) littermates were infected i.v. with 10^5^ PbA-infected red blood cells (iRBCs) at day 0. Absolute numbers of CD8^+^ T cells, CD11a^+^CD8^+^ T cells and GzmB^+^CD8^+^ T cells in (A) spleen and (B) blood were determined at day 6 or 7 after infection by flow cytometry. Data from 2–4 independent experiments with n = 13–22 mice in total (A) or from 2 experiments with n = 7–8 mice in total (B) are depicted as mean ± SD. Each dot represents one animal.
(TIF)

S7 Fig Expression of activation-associated molecules does not differ between stimulated CD8^+^ T cells from Nrp-1WT and Nrp-1KO mice. MACS-sorted CD8^+^ T cells from spleen of Nrp-1^fl/fl^ x CD4cre^wt^ (WT, black bars) and Nrp-1^fl/fl^ x CD4cre^tg^ (KO, white bars) littermates were stimulated *in vitro* with 1 μg/ml, 0.5 μg/ml or 0.1 μg/ml αCD3 plate-bound/αCD28 soluble for two days. The expression of CD69, CD44, PD-1, GzmB and Ki-67 was analyzed on gated CD8^+^ T cells by flow cytometry. Data from 2 (stimulation with 0.5 μg or 0.1 μg) or 4 (stimulation with 1 μg) independent experiments with n = 3 mice per experiment are summarized as mean ± SD. Each dot represents one animal. Statistical analysis was performed with one-way ANOVA. *, p<0.05; **, p<0.01; ****, p<0.0001.
(TIF)

S8 Fig Numbers of peripheral immune cells in the brain and manifestation of ECM are independent of Treg-specific Nrp-1 ablation. Nrp-1^fl/fl^ x Foxp3cre^tg^ (KO, white bars) and Nrp-1^+/+^ x Foxp3cre^tg^ littermates (WT, black bars) were infected i.v. with 10^5^ PbA-infected red blood cells (iRBCs) at day 0. (A) Numbers of CD45^high^ peripheral immune cells, CD4^+^ and CD8^+^ T cells were analyzed in the brain on day 6 or 7 after infection. (B) The severity of ECM was assessed by the RMCBS score and (C) brain weight was evaluated after cardiac perfusion on day 6 or 7 post infection. Data from three independent experiments with n = 13 mice per group are presented as mean values with SD. Statistical analysis was performed with (A) nonparametric Mann-Whitney test or (C) Student’s t test and for ECM Pathology and parasitemia with ordinary 2-way ANOVA and Sidak’s multiple comparisons test. *, p<0.05.
(TIF)
