# Supplementary figures and images for: Neuropilin-1 identifies a subset of highly activated CD8+ T cells during parasitic and viral infections
Source: PLoS Pathog. 2023 Nov 29;19(11):e1011837. doi: 10.1371/journal.ppat.1011837 (PMC10718454; doi:10.1371/journal.ppat.1011837)

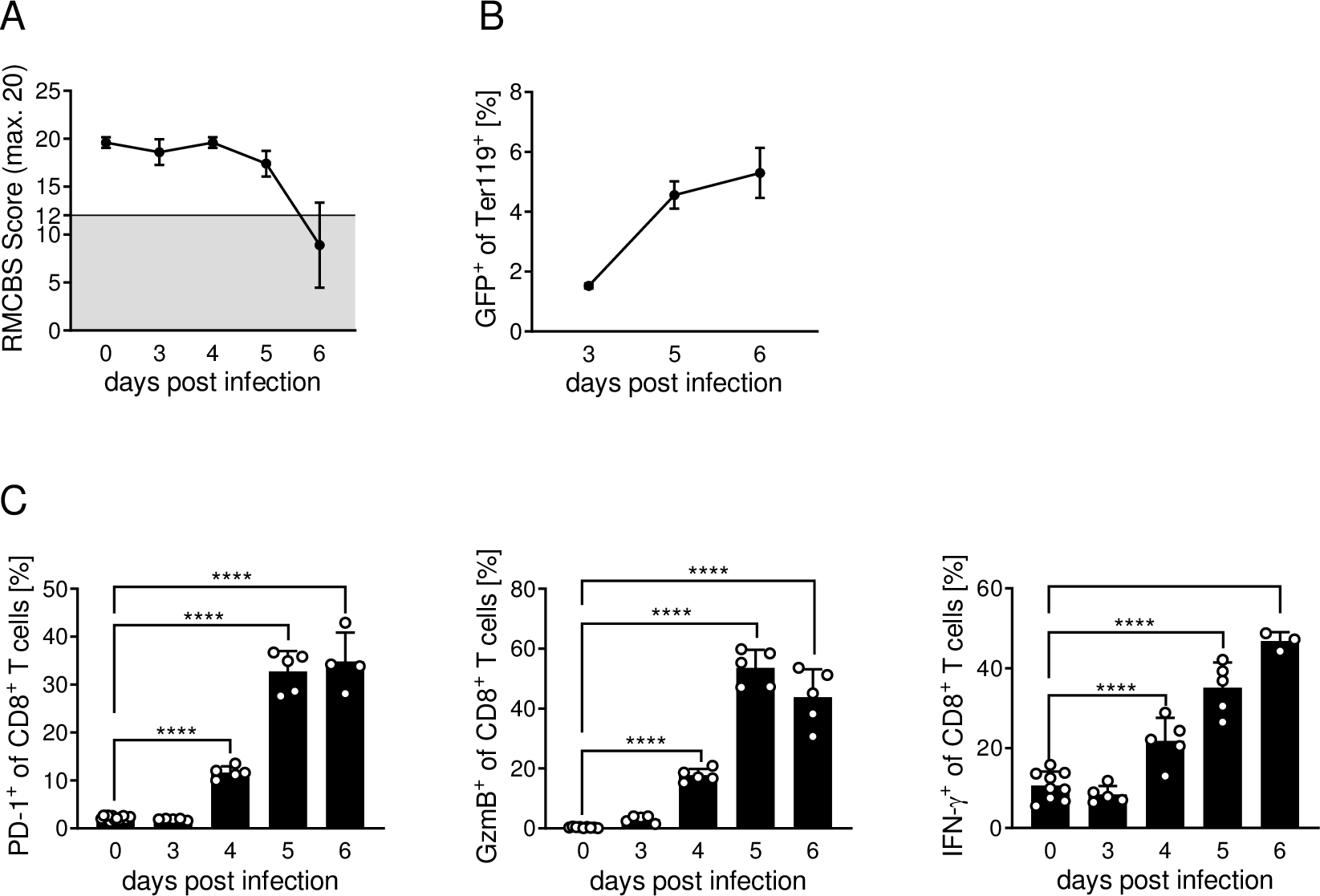

Supplement: S1 Fig — C57BL/6 mice were infected i.v. with 105 Plasmodium berghei ANKA (PbA) GFP+-infected erythrocytes. (A) The severity of experimental cerebral malaria (ECM) was assessed by the RMCBS score, which quantifies neurological deficits during development of ECM. Mice with a RMCBS score below 12 are considered to have ECM. (B) Parasitemia was determined by flow cytometry and calculated as the proportion of GFP+PbA-infected RBCs of total Ter119+ erythrocytes on day 3, 5 and 6 after PbA infection. (C) The frequency of PD-1-, GzmB- and IFN-γ-expressing CD8+ T cells was measured by flow cytometry in the spleen. Results from (A, B) n = 5 mice (d0, 3, 4, 5) from one experiment, n = 11 mice in total (d6) from two experiments, (C) n = 3–5 mice (d3, 4, 5, 6) from one experiment and n = 9–11 mice (d0) from three experiments are shown as mean + SD. Each dot represents one animal. Statistical significance was calculated with ordinary one-way ANOVA and Dunett`s multiple comparisons test. ***, p<0.001; ****, p<0.0001. (TIF) [file ppat.1011837.s001.tif]

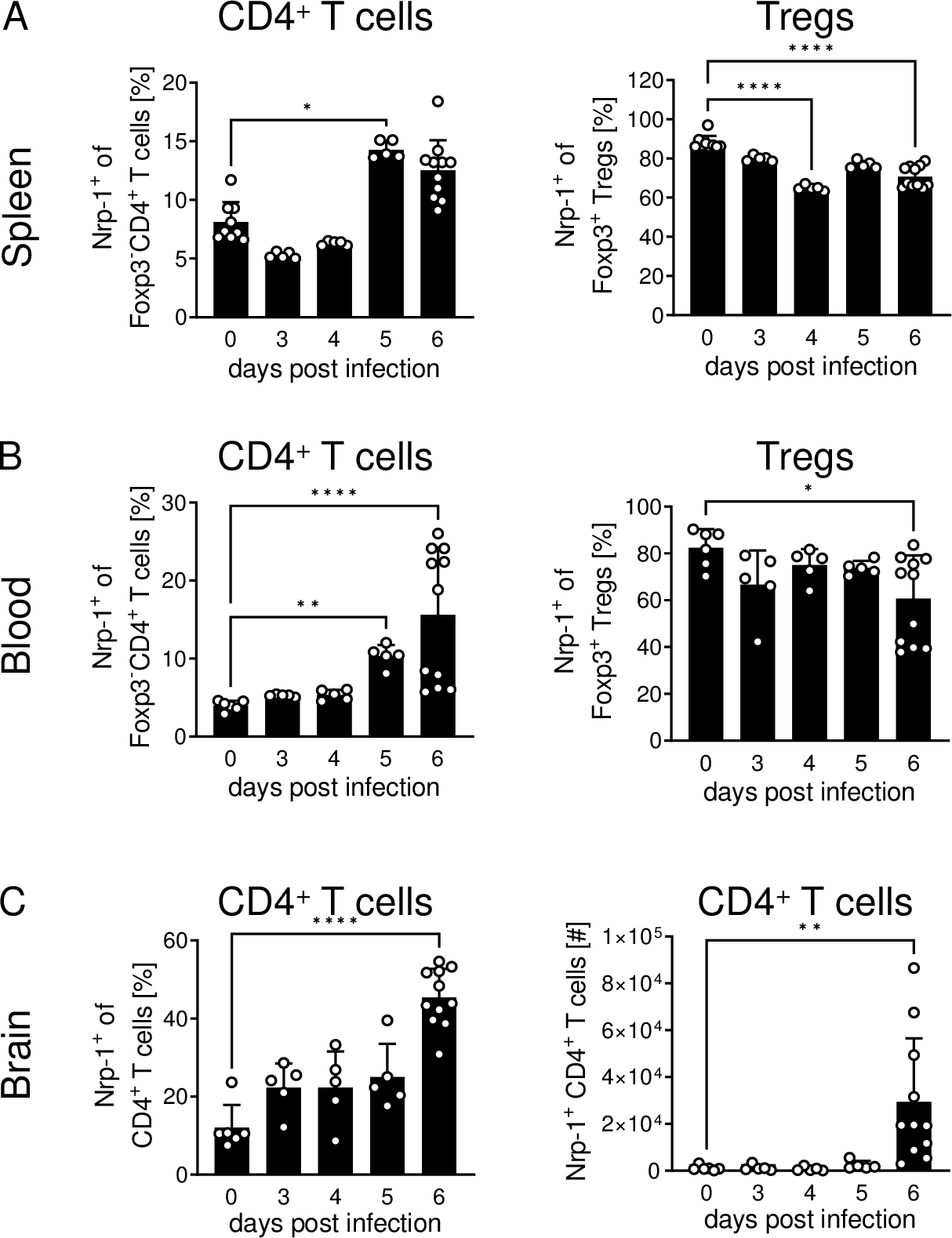

Supplement: S2 Fig — (A and B) Nrp-1 expression on CD4+Foxp3- conventional T cells and CD4+Foxp3+ Tregs was analyzed by flow cytometry on day 0 and in PbA-infected C57BL/6 mice at day 3, 4, 5 and 6 post infection in spleen and blood. (C) Frequencies and absolute numbers of peripheral CD4+ T cells of CD45high cells were measured in the brain by flow cytometry. Results from 1–2 independent experiments with n = 5–11 mice per time point are shown as mean + SD. Each dot represents one animal. Statistical significance was calculated with nonparametric Kruskal-Wallis test with Dunn’s multiple comparisons test. *, p<0.05; **, p<0.01; ****, p<0.0001. (TIF) [file ppat.1011837.s002.tif]

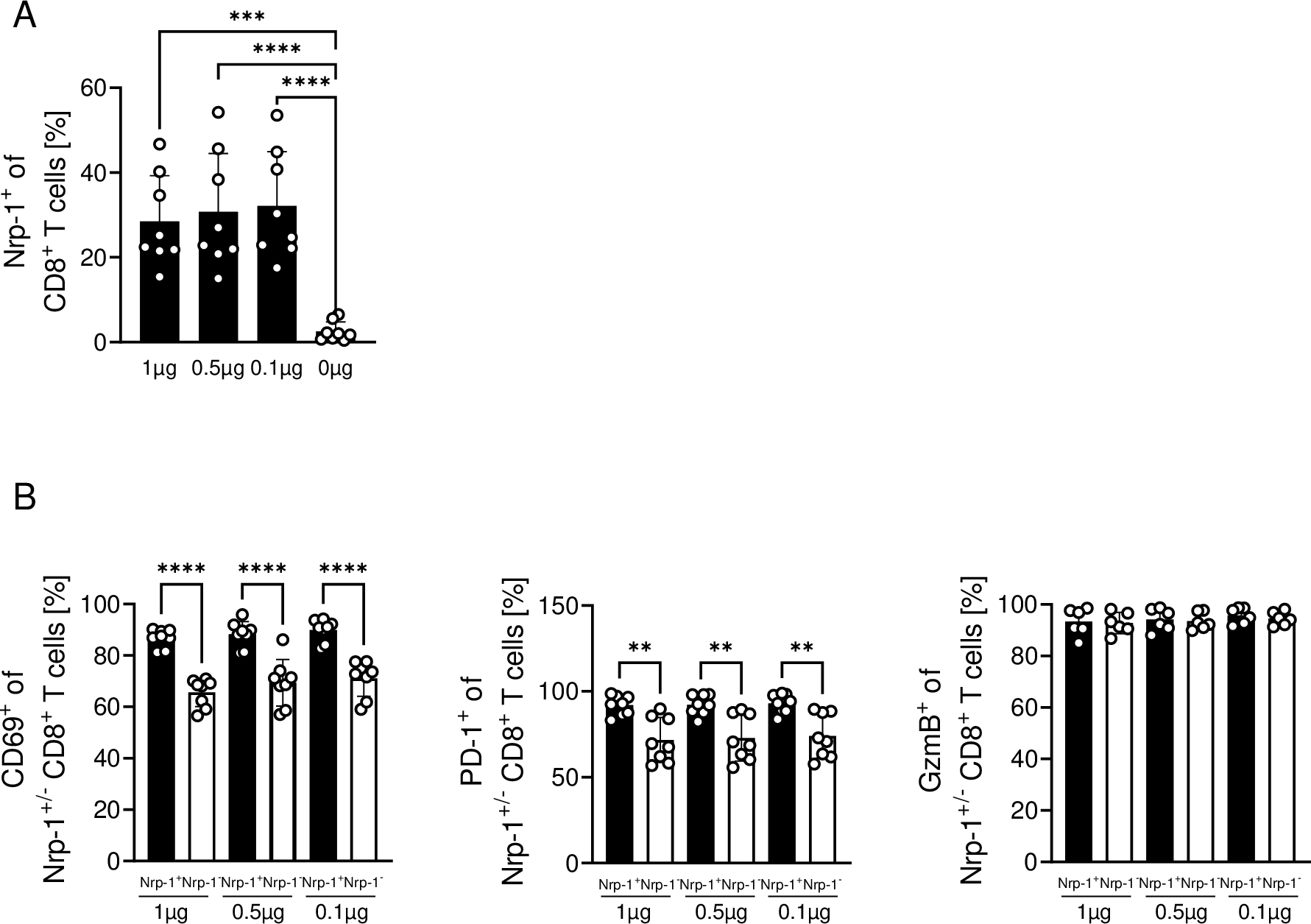

Supplement: S3 Fig — MACS-sorted CD8+ T cells isolated from spleen of OT-I mice were stimulated with indicated concentration of OVA in the presence of irradiated splenocytes as APCs for 48h. The expression of (A) Nrp-1 and (B) CD69, PD1 and GzmB on gated Nrp-1+CD8+ T cells and Nrp-1-CD8+ T cells was analyzed by flow cytometry. Results from three independent experiments with cells from n = 6–8 mice in total are summarized as mean ± SD. Statistical significance was calculated with one-way ANOVA and Tukey`s multiple comparisons test. **, p<0.01; ***, p<0.001; ****, p<0.0001. (TIF) [file ppat.1011837.s003.tif]

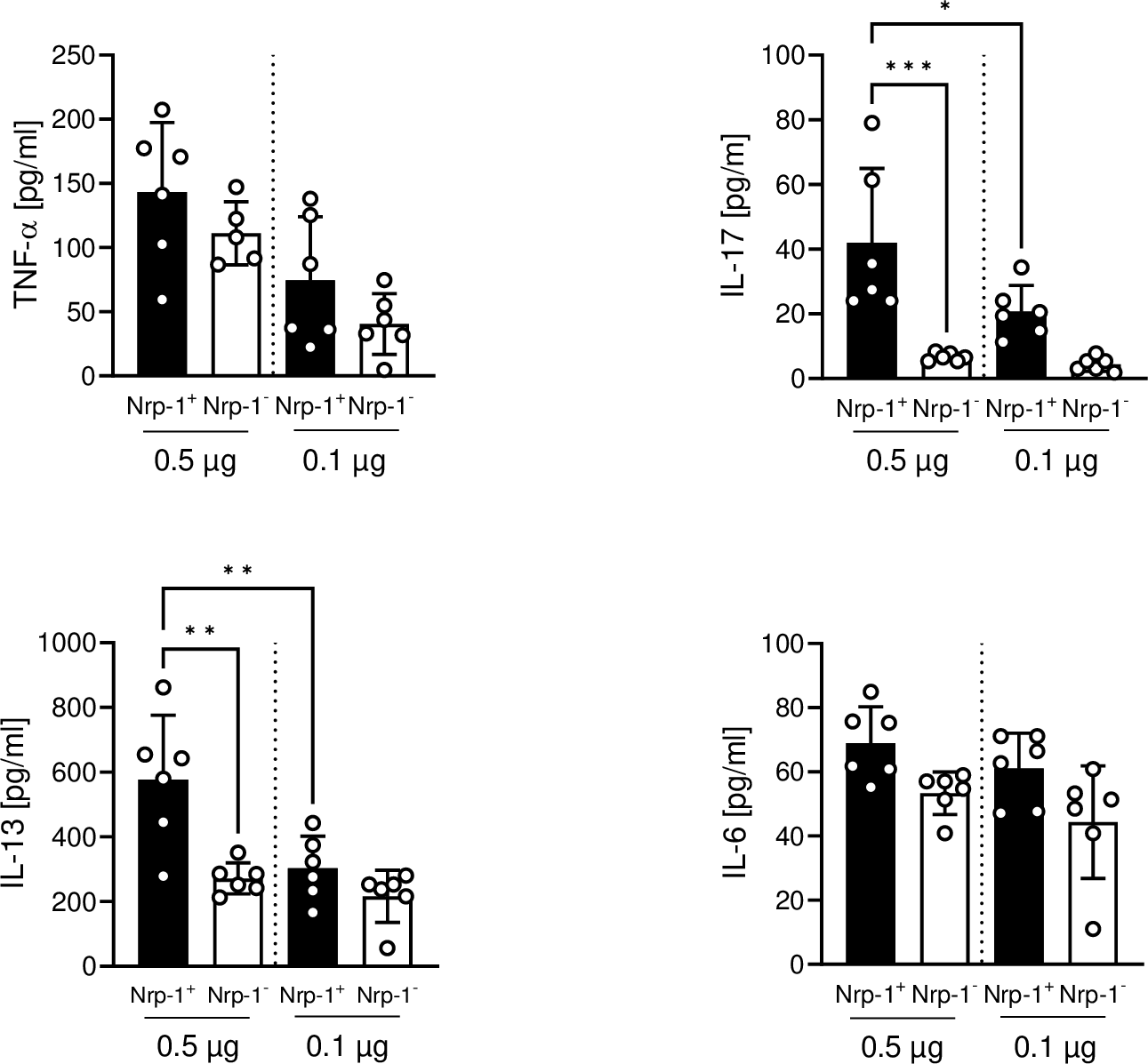

Supplement: S4 Fig — MACS-sorted CD8+ T cells from spleen of C57BL/6 mice were stimulated in vitro with 1 μg/ml αCD3/αCD28 for 48h. Nrp-1+CD8+ and Nrp-1-CD8+ T cells were sorted by FACS and re-stimulated with 0.5 μg/ml or 0.1 μg/ml αCD3 plate-bound/αCD28 soluble for another 48h. The concentration of cytokines in the supernatant was determined by Luminex technology. Data from 2 independent experiments with n = 3 mice per experiment are summarized as mean ± SD. Statistical analysis was performed with one-way ANOVA. *, p<0.05; **, p<0.01; ****, p<0.0001. (TIF) [file ppat.1011837.s004.tif]

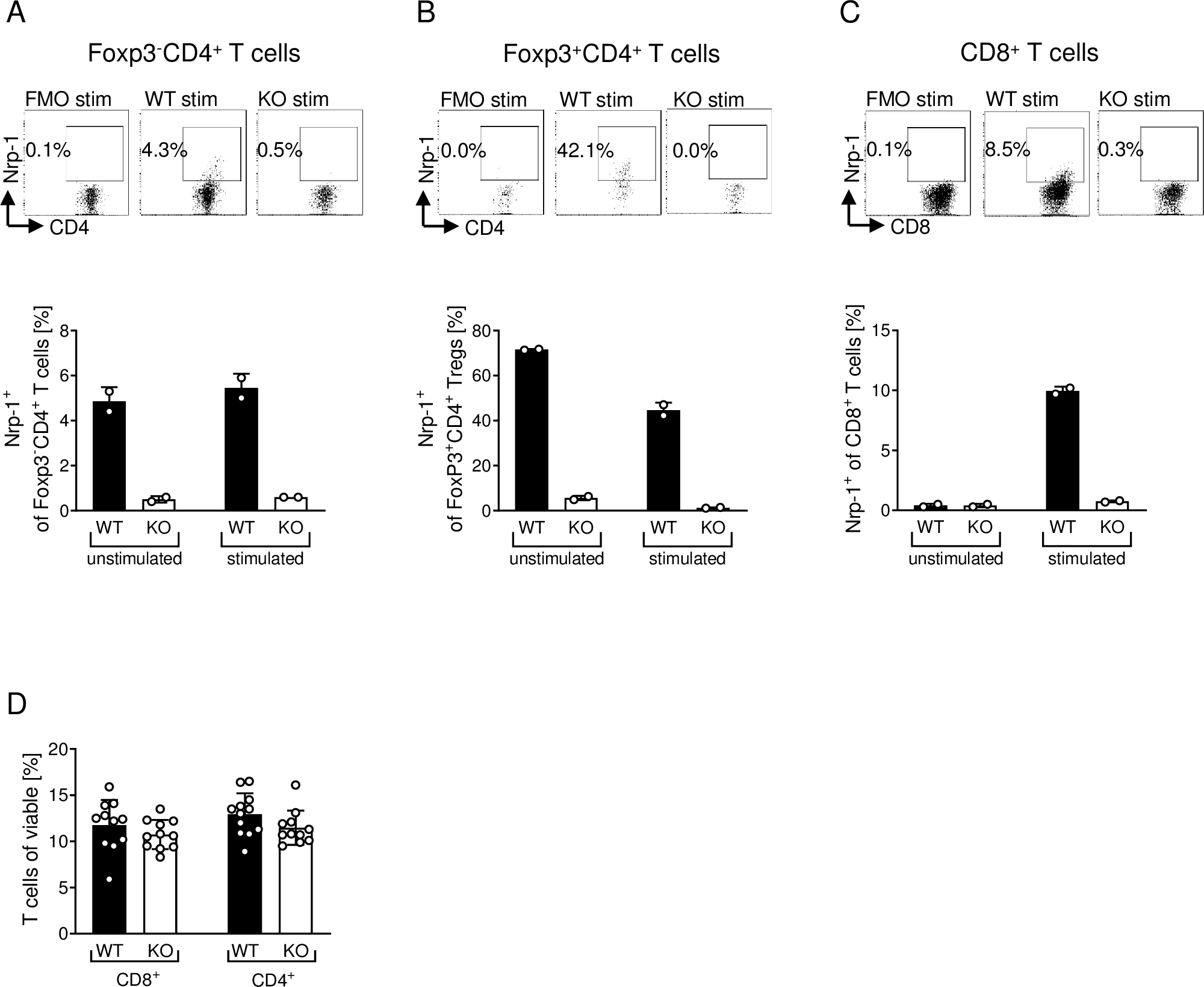

Supplement: S5 Fig — Splenocytes from Nrp-1fl/fl x CD4crewt (WT, black bars) and Nrp-1fl/fl x CD4cretg (KO, white bars) littermates were cultured in vitro (unstimulated) and stimulated with αCD3 and αCD28 (stimulated) for 48 hours. Successful ablation of Nrp-1 expression in (A) Foxp3-CD4+ conventional T cells, (B) Foxp3+CD4+ Tregs and (C) CD8+ T cells was verified by flow cytometry and is shown as mean ± SD. Representative FACS plots including FMO controls are shown in the upper panels. Data from one experiment with n = 2 mice is depicted. (D) The percentages of CD8+ and CD4+ T cells in spleen of naïve Nrp-1fl/fl x CD4crewt (WT) and Nrp-1fl/fl x CD4cretg (KO) littermates were determined by flow cytometry. Data from 2–3 independent experiments with n = 9–12 mice in total are shown as mean ± SD. Each dot represents one animal. (TIF) [file ppat.1011837.s005.tif]

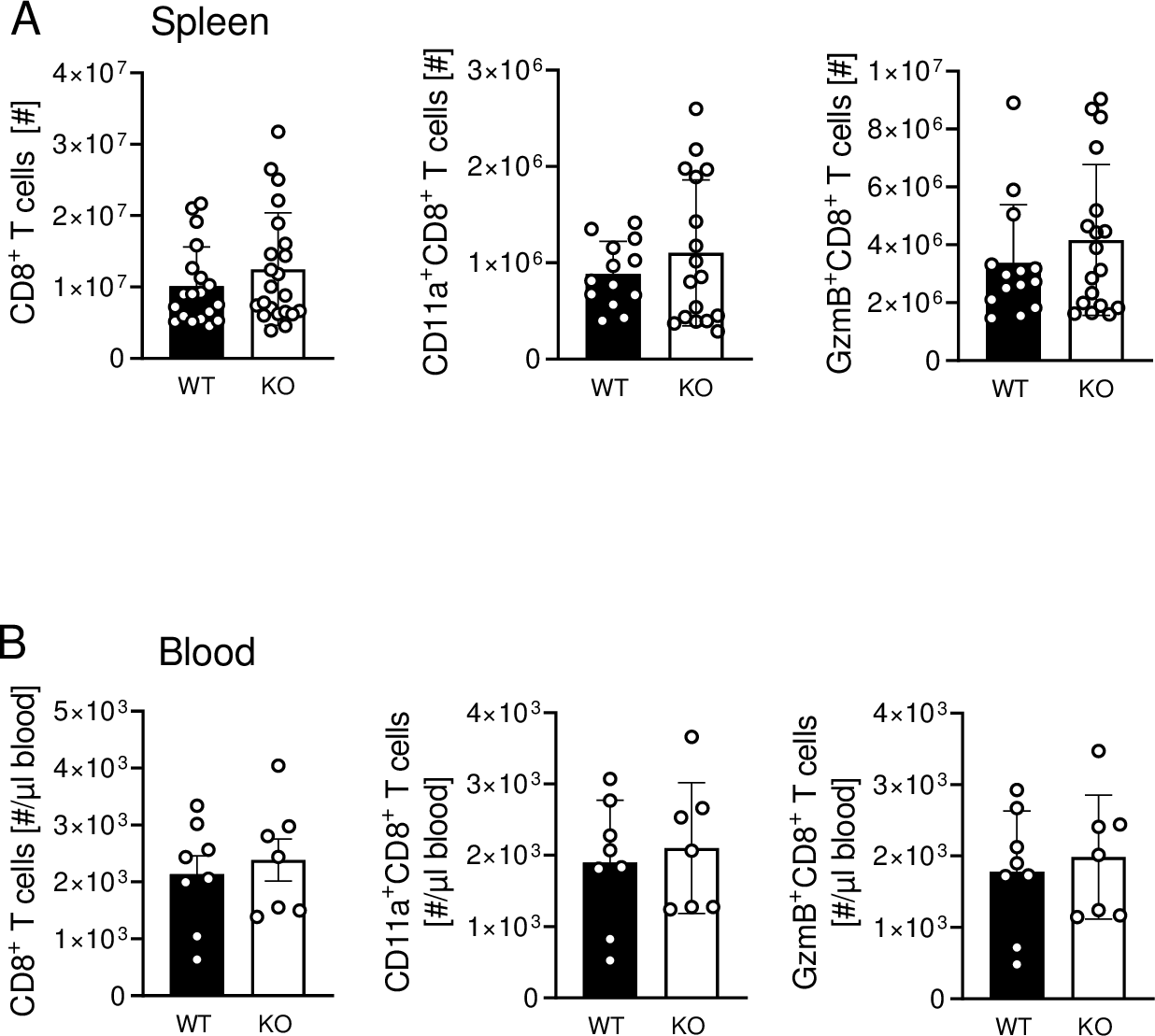

Supplement: S6 Fig — Nrp-1fl/fl x CD4crewt (WT, black bars) and Nrp-1fl/fl x CD4cretg (KO, white bars) littermates were infected i.v. with 105 PbA-infected red blood cells (iRBCs) at day 0. Absolute numbers of CD8+ T cells, CD11a+CD8+ T cells and GzmB+CD8+ T cells in (A) spleen and (B) blood were determined at day 6 or 7 after infection by flow cytometry. Data from 2–4 independent experiments with n = 13–22 mice in total (A) or from 2 experiments with n = 7–8 mice in total (B) are depicted as mean ± SD. Each dot represents one animal. (TIF) [file ppat.1011837.s006.tif]

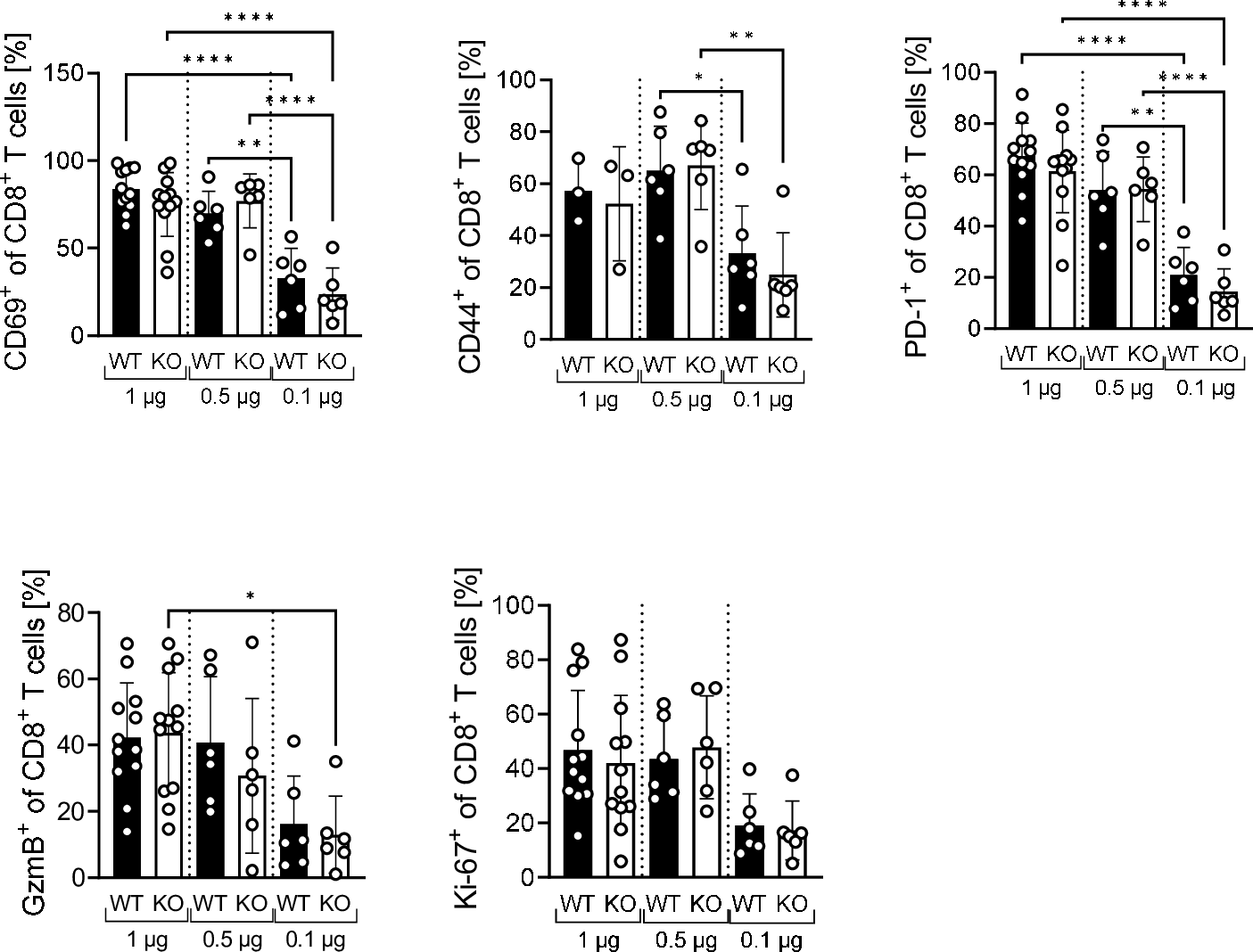

Supplement: S7 Fig — MACS-sorted CD8+ T cells from spleen of Nrp-1fl/fl x CD4crewt (WT, black bars) and Nrp-1fl/fl x CD4cretg (KO, white bars) littermates were stimulated in vitro with 1 μg/ml, 0.5 μg/ml or 0.1 μg/ml αCD3 plate-bound/αCD28 soluble for two days. The expression of CD69, CD44, PD-1, GzmB and Ki-67 was analyzed on gated CD8+ T cells by flow cytometry. Data from 2 (stimulation with 0.5 μg or 0.1 μg) or 4 (stimulation with 1 μg) independent experiments with n = 3 mice per experiment are summarized as mean ± SD. Each dot represents one animal. Statistical analysis was performed with one-way ANOVA. *, p<0.05; **, p<0.01; ****, p<0.0001. (TIF) [file ppat.1011837.s007.tif]

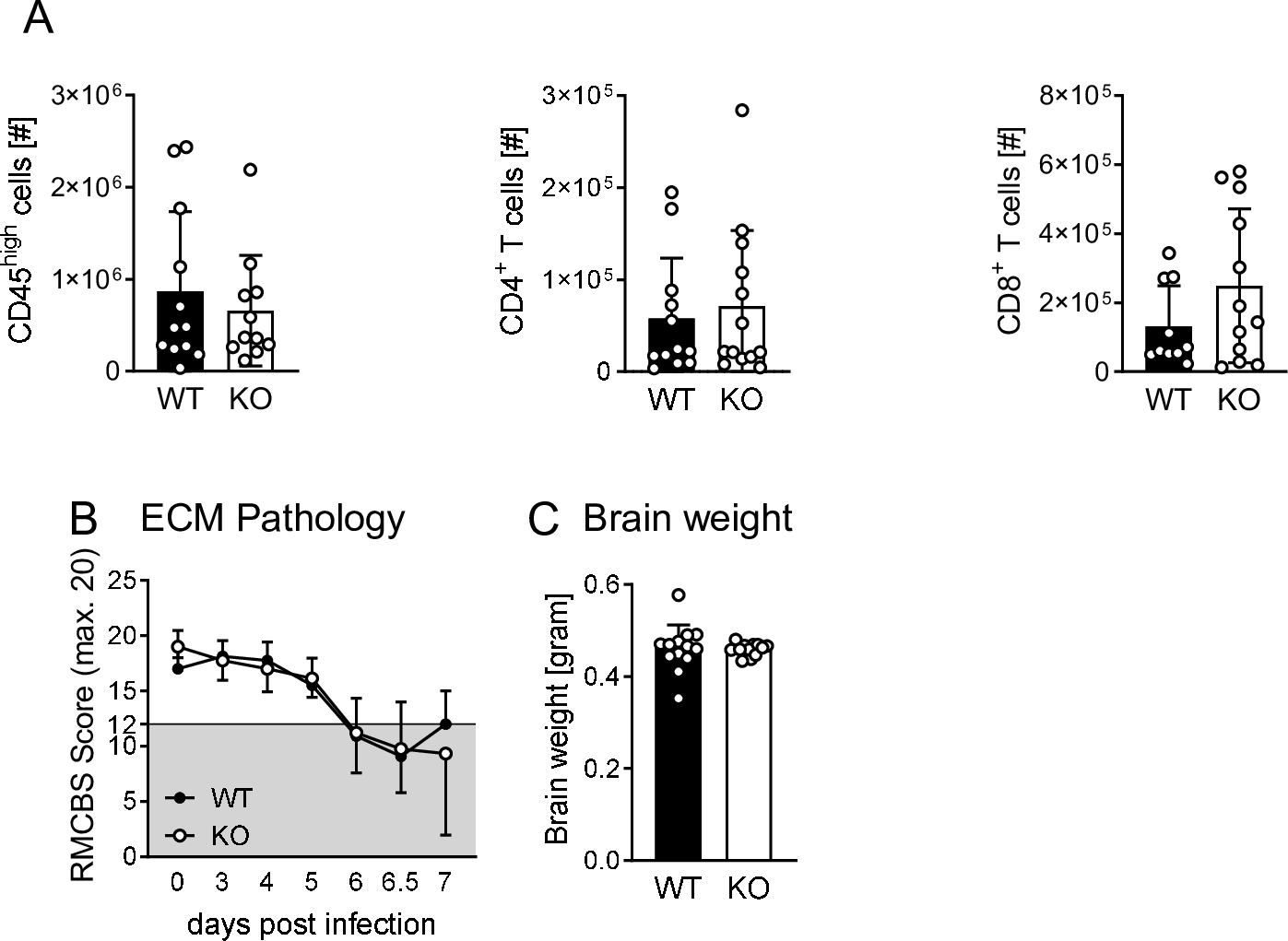

Supplement: S8 Fig — Nrp-1fl/fl x Foxp3cretg (KO, white bars) and Nrp-1+/+ x Foxp3cretg littermates (WT, black bars) were infected i.v. with 105 PbA-infected red blood cells (iRBCs) at day 0. (A) Numbers of CD45high peripheral immune cells, CD4+ and CD8+ T cells were analyzed in the brain on day 6 or 7 after infection. (B) The severity of ECM was assessed by the RMCBS score and (C) brain weight was evaluated after cardiac perfusion on day 6 or 7 post infection. Data from three independent experiments with n = 13 mice per group are presented as mean values with SD. Statistical analysis was performed with (A) nonparametric Mann-Whitney test or (C) Student’s t test and for ECM Pathology and parasitemia with ordinary 2-way ANOVA and Sidak’s multiple comparisons test. *, p<0.05. (TIF) [file ppat.1011837.s008.tif]
